# Macromitophagy is a longevity assurance process that in chronologically aging yeast limited in calorie supply sustains functional mitochondria and maintains cellular lipid homeostasis

**DOI:** 10.18632/aging.100547

**Published:** 2013-03-30

**Authors:** Vincent R. Richard, Anna Leonov, Adam Beach, Michelle T. Burstein, Olivia Koupaki, Alejandra Gomez-Perez, Sean Levy, Lukas Pluska, Sevan Mattie, Rami Rafeh, Tatiana Iouk, Sara Sheibani, Michael Greenwood, Hojatollah Vali, Vladimir I. Titorenko

**Affiliations:** ^1^ Department of Biology, Concordia University, Montreal, Quebec H4B 1R6, Canada; ^2^ Department of Anatomy and Cell Biology, McGill University, Montreal, Quebec H3A 2B2, Canada; ^3^ Department of Chemistry and Chemical Engineering, Royal Military College of Canada, Kingston, Ontario K7K 7B4, Canada

**Keywords:** yeast, mitophagy, mitochondria, cellular aging, caloric restriction, respiratory complexes and supercomplexes, lipid homeostasis

## Abstract

Macromitophagy controls mitochondrial quality and quantity. It involves the sequestration of dysfunctional or excessive mitochondria within double-membrane autophagosomes, which then fuse with the vacuole/lysosome to deliver these mitochondria for degradation. To investigate a physiological role of macromitophagy in yeast, we examined how the *atg32Δ*-dependent mutational block of this process influences the chronological lifespan of cells grown in a nutrient-rich medium containing low (0.2%) concentration of glucose. Under these longevity-extending conditions of caloric restriction (CR) yeast cells are not starving. We also assessed a role of macromitophagy in lifespan extension by lithocholic acid (LCA), a bile acid that prolongs yeast longevity under CR conditions. Our findings imply that macromitophagy is a longevity assurance process underlying the synergistic beneficial effects of CR and LCA on yeast lifespan. Our analysis of how the *atg32Δ* mutation influences mitochondrial morphology, composition and function revealed that macromitophagy is required to maintain a network of healthy mitochondria. Our comparative analysis of the membrane lipidomes of organelles purified from wild-type and *atg32Δ* cells revealed that macromitophagy is required for maintaining cellular lipid homeostasis. We concluded that macromitophagy defines yeast longevity by modulating vital cellular processes inside and outside of mitochondria.

## INTRODUCTION

Mitophagy is a key mechanism of mitochondrial quality and quantity control responsible for the autophagic degradation of aged, dysfunctional, damaged or excessive mitochondria [1-3]. A micromitophagic mode of mitophagy involves the engulfment of such mitochondria through direct invagination of the vacuolar/lysosomal boundary membrane [4, 5], whereas its macromitophagic mode refers to the sequestration of targeted mitochondria into double-membrane-bounded structures known as autophagosomes [2, 5]. Following fusion of these autophagosomes with the vacuole/ lysosome, sequestered mitochondria are degraded by acid hydrolases [1, 6].

Only the macromitophagic mode of mitophagy has been described in mammals [7, 8]. Macromitophagy in mammalian cells is known to play essential roles in several vital biological processes underlying organismal aging, development and differentiation, including (i) selective degradation of depolarized mitochondria in dopaminergic neurons in the substantia nigra, a PINK1/Parkin-dependent process impaired in autosomal recessive forms of Parkinson's disease; (ii) massive elimination of mitochondria driven by Nix, a protein in the outer mitochondrial membrane, during reticulocyte-to-erythrocyte maturation; and (iii) selective clearance of surplus mitochondria during white adipose tissue differentiation in an Atg5/Atg7-dependent manner [2, 3, 7-9].

The physiological role(s) of mitophagy in yeast remain(s) obscure. Exposure to rapamycin, starvation and mutations causing mitochondrial depolarization, swelling, fragmentation or biogenesis defects trigger a Uth1p-dependent process of micromitophagy in yeast cells cultured on lactate, a non-fermentable carbon source [4, 5].

In yeast grown on lactate, an Aup1p-dependent process of macromitophagy has been proposed to maintain cell survival following entry into a quiescent state [10]. Aup1p is a mitochondrial phosphatase. In pre-quiescent cells, it is abundant and confined to the intermembrane space in functional mitochondria organized into a network [10]. However, the level of Aup1p is significantly diminished in cells that entered quiescence [10]. This reduction in the cellular level of Aup1p during stationary phase coincides with mitochondrial network fragmentation in quiescent yeast, in which Aup1p is mainly cytosolic [10]. Based on these findings, it has been suggested that macromitophagy is an essential pro-survival process in yeast cells that entered quiescence following growth under conditions requiring a large numbers of functional mitochondria [10]. Noteworthy, Aup1p has been shown to promote the dephosphorylation and nuclear import of Rtg3p, a key transcriptional activator of the mitochondrial retrograde (RTG) signaling pathway [11]. The RTG pathway is one of the major longevity-defining signaling pathways in yeast cells; it governs transcription of numerous genes that regulate longevity by modulating carbohydrate and nitrogen metabolism, peroxisome proliferation, peroxisomal fatty acid ß-oxidation and anaplerotic reactions, stress responses, and the stability of nuclear and mitochondrial genomes [12]. Furthermore, Aup1p is also known to dephosphorylate and activate Pda1p, the α-subunit of the mitochondrial pyruvate dehydrogenase complex essential for the tricarboxylic acid cycle, respiration and nitrogen assimilation in mitochondria [13]. Therefore, it is plausible that the demonstrated pro-survival function of Aup1p in yeast cells grown under respiratory conditions [10] does not necessarily imply that macromitophagy is essential for maintaining survival of these cells following entry into quiescence, but rather reflects the essential roles of Aup1p in (i) maintaining a network of polarized, functional mitochondria during stationary phase; (ii) activating the RTG signaling pathway that governs numerous longevity-defining processes outside of mitochondria; and (iii) sustaining high enzymatic activity of Pda1p, thereby supporting vital mitochondrial metabolic processes known to regulate yeast longevity.

In yeast pre-cultured on lactate and then shifted to a medium that contains glucose (a fermentable carbon source) and lacks nitrogen source, macromitophagy eliminates mitochondria to minimize reactive oxygen species (ROS) formation and reduce the resulting mitochondrial DNA damage [14]. Atg32p has been shown to function as a mitochondrial receptor for selective macroautophagic removal of mitochondria under these nitrogen starvation conditions, as well as in yeast cells entered a quiescent state under respiratory conditions in lactate-based medium [15, 16]. Atg32p binding to an adaptor protein Atg11p is known to drive the recruitment of mitochondria to the phagophore assembly site, thereby initiating the macromitophagy process [15, 16]. In addition to Atg32p, numerous other proteins involved in this selective process have been identified in genome-wide screens [16, 17]. It needs to be emphasized that *atg32Δ* cells cultured in a non-fermentable medium do not exhibit any growth or physiological defects indicative of mitochondrial dysfunction [16, 17]. Thus, the physiological role of Atg32p-driven macromitophagy in yeast remains to be established.

To provide further insight into the physiological functions of selective macroautophagic mitochondrial removal in the yeast *Saccharomyces cerevisiae*, in this study we examined how the *atg32Δ*-dependent mutational block of macromitophagy affects the chronological lifespan of yeast cultured in a nutrient-rich medium initially containing low (0.2%) concentration of glucose. It has been shown that under these longevity-extending CR conditions yeast cells limited in calorie supply are not starving but undergo an extensive remodeling of their metabolism in order to match the level of ATP produced under longevity-shortening non-CR conditions [18]. Our recent data also demonstrated that LCA, a bile acid, is a potent anti-aging natural compound that acts in synergy with CR to enable a significant further extension of yeast lifespan under CR conditions [19]. Therefore, in this study we evaluated the role of macromitophagy in yeast lifespan extension by LCA. Furthermore, we also examined how the *atg32Δ* mutation influences mitochondrial morphology, protein and lipid compositions, oxidative damage, and function. Moreover, because mitochondria are known to be dynamically integrated into a network governing lipid metabolism and transport not only within these organelles but also within the endoplasmic reticulum (ER) and the plasma membrane (PM) [20-27], in this study we used mass spectrometry to compare the membrane lipidomes of these organelles purified from wild-type (WT) and *atg32Δ* cells that were cultured under CR on 0.2% glucose. Our findings provide evidence that macromitophagy defines longevity of chronologically aging yeast limited in calorie supply, underlies the synergistic beneficial effects of CR and LCA on lifespan, modulates a compendium of vital processes confined to mitochondria, and maintains cellular lipid homeostasis.

## RESULTS

### Macromitophagy is a longevity assurance process

**Figure 1:**
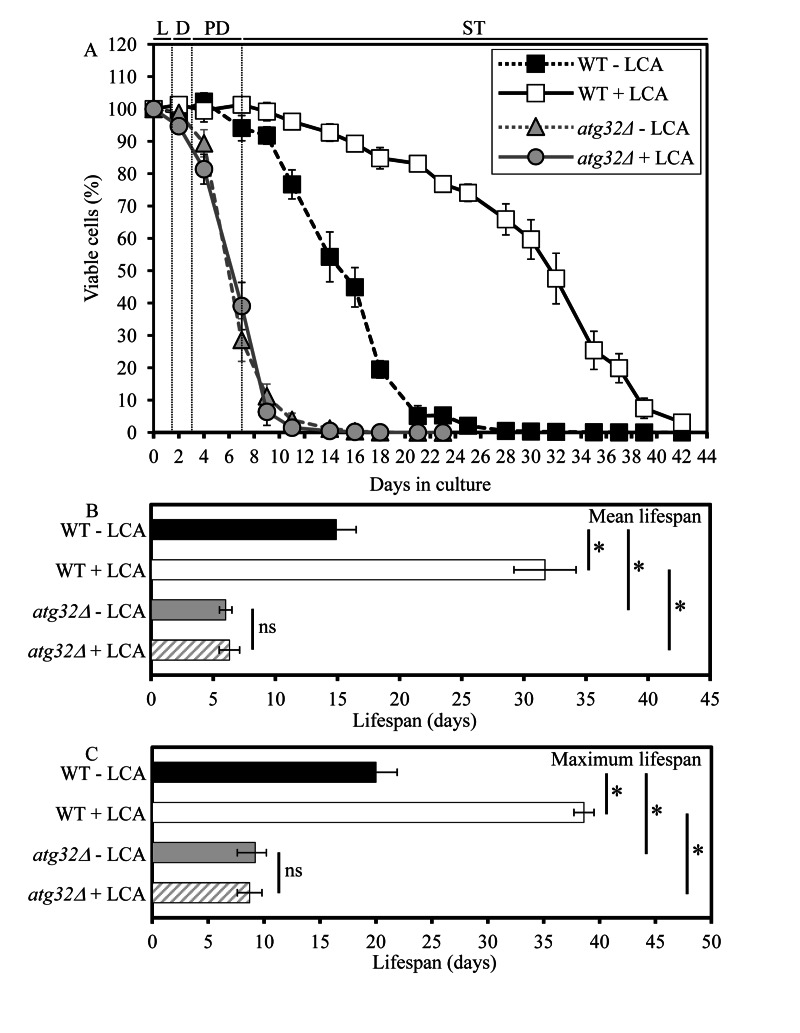
Under CR conditions, the *atg32Δ*-dependent mutational block of macromitophagy substantially shor-tens yeast CLS and abolishes the longevity-extending effect of LCA WT and *atg32Δ* strains were cultured in the nutrient-rich YP medium initially containing 0.2% glucose in the presence or absence of 50 μM LCA. Effect of the*atg32Δ* mutation and LCA on survival (**A**) and on the mean (**B**) and maximum (**C**) lifespans of chronologically aging yeast grown under CR conditions on 0.2% glucose. Data are presented as means ± SEM (n = 6-8; *p < 0.01). Abbreviations: Diauxic (D), logarithmic (L), post-diauxic (PD) or stationary (ST) growth phase.

As a first step towards addressing a role of selective macroautophagic mitochondrial removal in sustaining essential biological processes in yeast, we evaluated the importance of macromitophagy in longevity assurance. We compared the chronological lifespan (CLS) of wild-type (WT) strain to that of the single-gene-deletion mutant strain *atg32Δ*, which is impaired only in the mitophagic pathway of selective macroautophagy but not in other pathways of selective or non-selective macroautophagy [15, 16]. Both yeast strains were cultured in the nutrient-rich YP (1% yeast extract and 2% peptone) medium initially containing low (0.2%) concentration of glucose, a fermentable carbon source. Importantly, yeast cells grown under these longevity-extending caloric restriction (CR) conditions are not starving [18]. We found that the *atg32Δ* mutation substantially shortens both the mean and maximum CLS of yeast limited in calorie supply (Figure 1). Thus, macromitophagy is an essential longevity assurance process in chronologically aging yeast grown under CR on 0.2% glucose.

**Figure 2:**
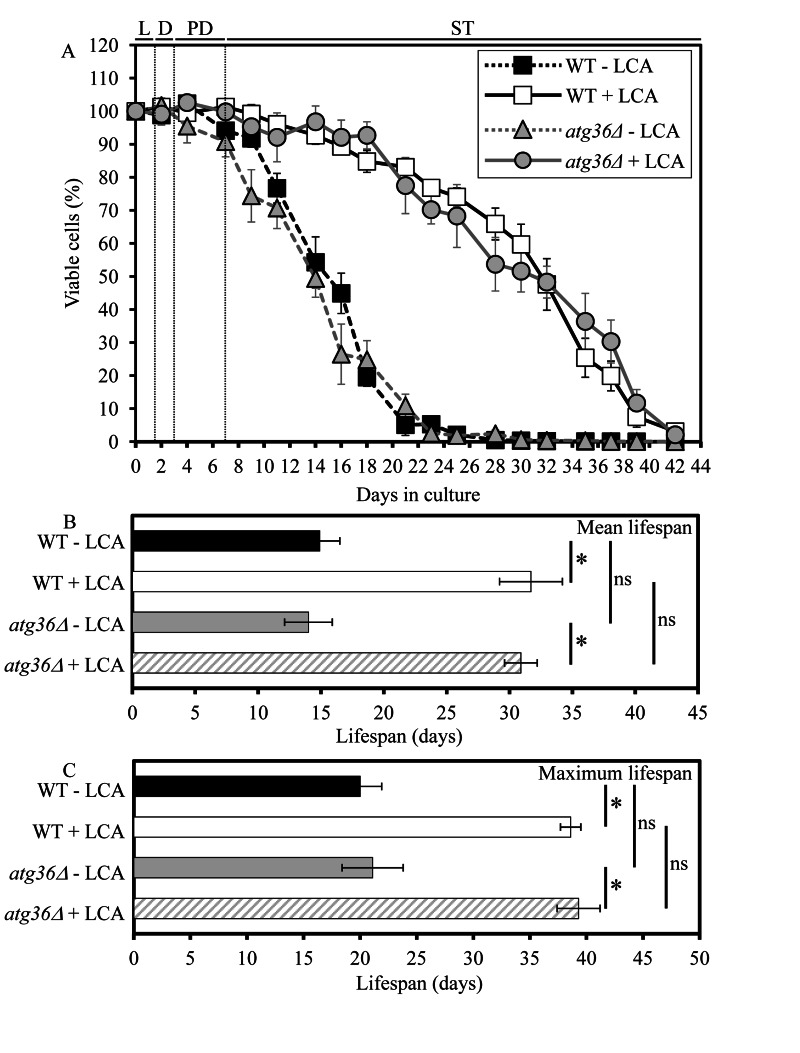
Under CR conditions, the *atg36Δ*-dependent mutational block of macropexophagy does not alter yeast CLS and does not compromise the longevity-extending efficacy of LCA WT and *atg36Δ* strains were cultured in the nutrient-rich YP medium initially containing 0.2% glucose in the presence or absence of 50 μM LCA. Effect of the*atg36Δ* mutation and LCA on survival (**A**) and on the mean (**B**) and maximum (**C**) lifespans of chronologically aging yeast grown under CR conditions on 0.2% glucose. Data are presented as means ± SEM (n = 5-6; *p < 0.01; ns, not significant). Abbreviations: Diauxic (D), logarithmic (L), post-diauxic (PD) or stationary (ST) growth phase.

The essential role of macromitophagy in regulating yeast longevity under CR conditions is limited to this form of selective macroautophagy. Indeed, the single-gene-deletion mutation *atg36Δ*, known to impair only the pexophagic pathway of selective macroautophagy but not mitophagic or any other pathways of selective or non-selective macroautophagy [28], did not influence the mean or maximum CLS of yeast limited in calorie supply (Figure 2).

### Macromitophagy is required for longevity extension by an anti-aging compound

Our recent study revealed that lithocholic acid (LCA), a bile acid, extends the CLS of yeast grown under CR on 0.2% glucose [19]. To assess a role of selective macroautophagic mitochondrial removal in longevity extension by LCA, we tested if this bile acid added to growth medium at the time of cell inoculation is able to extend longevity of the macromitophagy-deficient *atg32Δ* mutant. As we found, the *atg32Δ* mutation abolishes the ability of LCA to increase both the mean and maximum CLS of yeast limited in calorie supply (Figure 1). Hence, macromitophagy is required for longevity extension by LCA in chronologically aging yeast grown under CR on 0.2% glucose.

Unlike macromitophagy, the pexophagic form of selective macroautophagy is not obligatory for the ability of LCA to extend longevity of chronologically aging yeast under CR conditions. In fact, the exclusive impairment of macropexophagy by the single-gene-deletion mutation *atg36Δ* did not compromise the longevity-extending efficacy of LCA in yeast limited in calorie supply (Figure 2).

### Macromitophagy defines the size and number of mitochondria, their shape and morphology, and their ability to exist as a network

**Figure 3:**
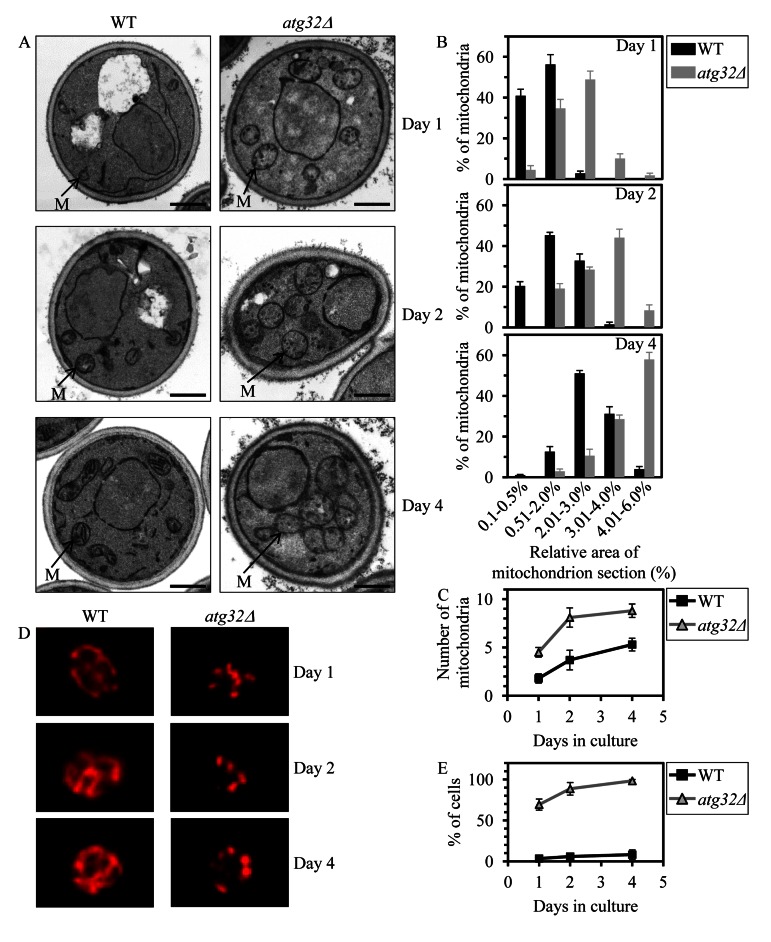
Under CR conditions,the *atg32Δ*-dependent mutational block of macromito-phagy alters the age-related dynamics of changes in mitochondrial size, number, shape, morphology and network appearance WT and *atg32Δ* strains were cultured in the nutrient-rich YP medium initially containing 0.2% glucose. (**A**) Transmission electron micrographs of WT and *atg32Δ* cells recovered on day 1, 2 or 4 of cell culturing. M, mitochondrion. Bar, 1 μm. (**B**) Percentage of mitochondria in WT and *atg32Δ* cells having the indicated relative area of mitochondrion section. The relative area of mitochondrion section was calculated as (area of mitochondrion section/area of cell section) × 100. Data are presented as means ± SEM (at least 100 cells of each strain were used for morphometric analysis at each time-point). (**C**) Numbers of mitochondria in WT and *atg32Δ* cells. The data of morphometric analysis are expressed as the number of mitochondria per μm^3^ of cell section ± SEM (at least 100 cells of each strain were used for morphometric analysis at each time-point). (**D**) Morphology of mitochondria in WT and *atg32Δ* cells recovered on day 1, 2 or 4 of cell culturing. Mitochondria were visualized by indirect immunofluorescence microscopy using monoclonal anti-porin primary antibodies and Alexa Fluor 568-conjugated goat anti-mouse IgG secondary antibodies. (**E**) The percentage of cells exhibiting fragmented mitochondria was calculated. At least 800 cells of each strain were used for quantitation at each time-point. Data are presented as mean ± SEM (n = 3).

To provide a mechanistic insight into the demonstrated essential role of macromitophagy in defining longevity of chronologically aging yeast under CR conditions, we used electron microscopy (EM) to compare the age-related dynamics of changes in the size, number and morphology of mitochondria in WT cells to that in macromitophagy-deficient *atg32Δ* cells. We found that in cells grown under CR on 0.2% glucose and recovered at different periods of CLS, the *atg32Δ* mutation (i) results in accumulation of greatly enlarged mitochondria (Figures 3A and 3B); (ii) substantially increases the number of mitochondria (Figures 3A and 3C); (iii) alters mitochondrial morphology by elevating the proportion of round-shaped mitochondria, especially in cells entered post-diauxic (PD) growth phase and recovered on day 4 of cell culturing (Figure 3A); and (iv) reduces the length of mitochondrial cristae and alters their morphological appearance (Figure 3A). Furthermore, our indirect immunofluorescence microscopy analysis of cells grown under CR on 0.2% glucose and recovered at different periods of CLS revealed that the *atg32Δ* mutation also causes massive fragmentation of the elaborate mito-chondrial network seen in WT cells (Figures 3B and 3E).

In sum, our microscopical analyses suggest that under CR conditions macromitophagy is required to maintain a network of mitochondria, perhaps by selectively eliminating individual mitochondria segregated from this network due to certain changes in the morphology of their cristae. One could envisage that the inability of macromitophagy-deficient *atg32Δ* cells to eliminate these morphologically distinct (and possibly dysfunctional) mitochondria following their segregation from the mitochondrial network may result in their progressive accumulation with age, thereby leading to the establishment of a pro-aging cellular pattern and ultimately shortening yeast longevity.

### Macromitophagy sustains a healthy population of functional mitochondria

**Figure 4:**
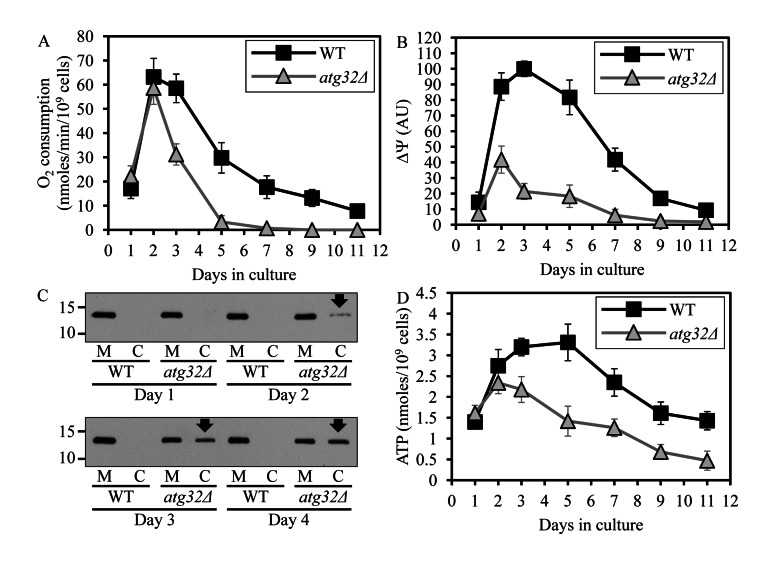
Under CR conditions, the *atg32Δ*-dependent mutational block of macromitophagy alters the age-related chronology of changes in vital mitochondrial functions WT and *atg32Δ* strains were cultured in the nutrient-rich YP medium initially containing 0.2% glucose. Effect of the*atg36Δ* mutation on the rate of oxygen consumption by cells (**A**), electrochemical potential across the inner mitochondrial membrane IMM (ΔΨ) (**B**), level of cytochrome *c* in purified mitochondria (M) and the cytosolic fraction (C) (**C**), and cellular level of ATP (**D**). Data in **A**, **B** and **D** are presented as means ± SEM (n = 6-9).

Based on our finding that macromitophagy plays an essential role in preserving the mitochondrial network in CR yeast, we hypothesized that this form of selective macroautophagy is required for sustaining a healthy population of functional mitochondria in chronologically aging cells. To test the validity of our hypothesis, we assessed the age-related dynamics of changes in several vital mitochondrial functions during chronological aging of WT and *atg32Δ* yeast cells. We found that in cells cultured under CR on 0.2% glucose and recovered at different periods of CLS, the *atg32Δ* mutation (i) leads to accumulation of dysfunctional mitochondria exhibiting reduced respiration (Figure 4A); (ii) causes a build-up of dysfunctional mitochondria having a reduced electrochemical potential across the inner mitochondrial membrane (IMM) (Figure 4B); (iii) triggers a release of cytochrome *c* from the intermediate space of mitochondria into the cytosol (Figure 4C), known to be a hallmark event of mitochondrial outer membrane permeabilization (MOMP) [29]; and (iv) reduces the cellular level of ATP (Figure 4D), which under CR conditions is produced mainly in mitochondria [30]. All these changes in vital mitochondrial functions were observed in macromitophagy-deficient *atg32Δ* cells as early as after 2 days of culturing (Figure 4).

These findings suggest that under CR conditions macromitophagy is required to sustain a healthy network of functional mitochondria that actively respire, exhibit high electrochemical potential across the IMM, maintain outer mitochondrial membrane impermeability to proteins and efficiently synthesize ATP. Thus, it is conceivable that in chronologically aging yeast limited in calorie supply macromitophagy selectively eliminates dysfunctional mitochondria impaired in vital mitochondrial functions that define longevity.

### Macromitophagy maintains a population of mitochondria whose inner membrane exhibits abundant respiratory and non-respiratory protein supercomplexes of distinct compositions

**Figure 5:**
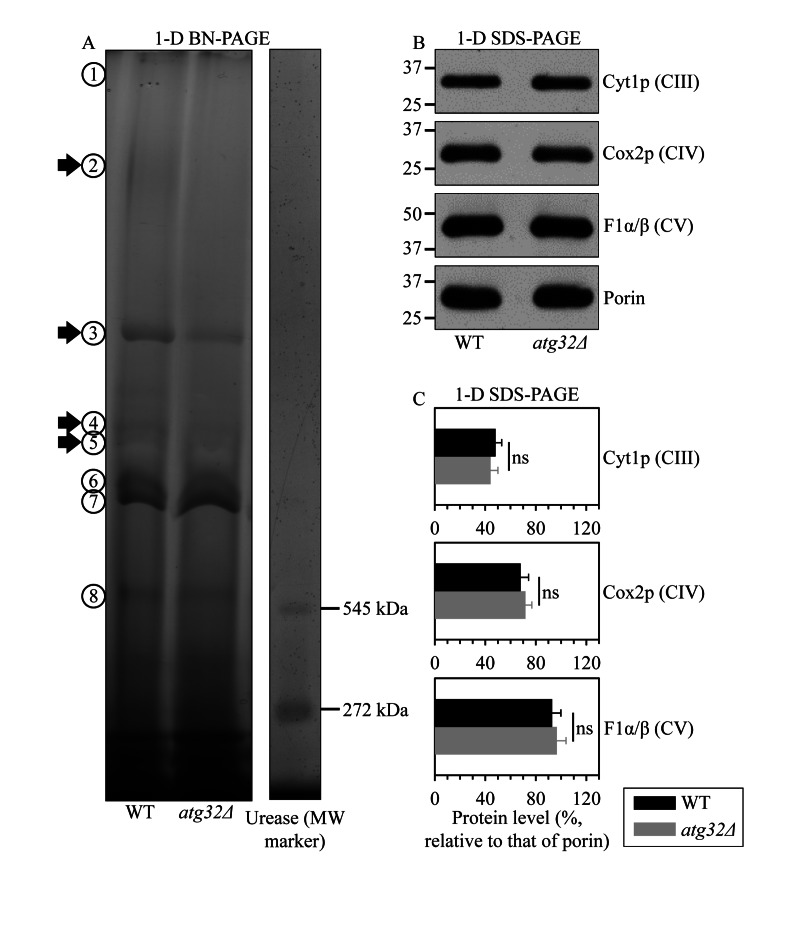
Under CR conditions, the *atg32Δ* mutation reduces the levels of several large protein super-complexes in the IMM, likely due to their dissociation into individual proteins or small protein subcomplexes WT and *atg32Δ* strains were cultured in the nutrient-rich YP medium initially containing 0.2% glucose. Mitochondria were purified from WT and *atg32Δ* cells recovered on day 4 of culturing. (**A**) Digitonin-solubilized protein complexes from the inner membrane of these mitochondria were separated on a linear 4-13% acrylamide gradient gel for first-dimension blue native PAGE (1-D BN-PAGE). Arrows mark the positions of protein supercomplexes 2, 3, 4 and 5 whose levels were reduced in the IMM of *atg32Δ* cells. (**B** and **C**) Equal quantities (10 μg) of protein from these mitochondria were subjected to first-dimension SDS-PAGE (1-D SDS-PAGE) and analyzed by quantitative immunoblotting with antibodies to porin (loading control), Cyt1p (cytochrome c1, a component of the respiratory complex III), Cox2p (subunit II of cytochrome c oxidase, a component of the respiratory complex IV) or F1α/β (alpha and beta subunits of the mitochondrial F1ATPase, the respiratory complex V). Data in **C** are presented as means ± SEM (n = 3-4; ns, not significant).

A body of recent evidence supports the view that, instead of existing as separate units freely moving within the IMM, individual respiratory protein complexes are assembled into supramolecular structures known as respiratory supercomplexes or respirasomes [31, 32]. Some changes in such supramolecular organization of respiratory protein complexes may cause detrimental alterations of the mitochondrial respiratory chain, thereby eliciting certain age-related pathologies [33]. We therefore hypothesized that the observed inability of prematurely aging under CR conditions *atg32Δ* cells to sustain a healthy population of functional mitochondria (Figure 4) may be caused in part by some changes in the abundance and/or composition of certain respiratory protein super-complexes. To validate this hypothesis, we purified mitochondria from WT and *atg32Δ* cells, used digitonin to solubilize protein complexes from the inner membrane of these mitochondria, and separated the solubilized protein complexes on a linear 4-13% acrylamide gradient gel for first-dimension blue native PAGE (1-D BN-PAGE). This gradient gel is known to resolve protein complexes within the mass range of 100 kDa - 3 MDa [34]. Our 1-D BN-PAGE analysis revealed that in cells cultured under CR on 0.2% glucose and recovered on day 4 of culturing, the *atg32Δ* mutation reduces the levels of several protein complexes in the IMM, including complexes 2, 3, 4 and 5 (Figure 5A). Because the molecular masses of all these and other protein complexes resolved by 1-D BN-PAGE exceeded 545 kDa, a molecular mass of the largest commercially available urease tetramer marker protein (Figure 5A), they represent very large protein supercomplexes assembled from several protein complexes [35]. Noteworthy, our separation of denatured monomeric membrane proteins from purified mitochondria by SDS-PAGE and their subsequent quantitative analysis by immunoblotting revealed that the *atg32Δ* mutation does not cause significant changes to the total levels of the respiratory complexes III, IV or V (Figures 5B and C). Hence, the observed reduction of the levels of protein supercomplexes 2, 3, 4 and 5 in the IMM of *atg32Δ* cells (Figure 5A) was not due to a decrease in the total amounts of the respiratory complexes III, IV or V. It is conceivable therefore that in the IMM of *atg32Δ* cells a significant portion of these protein supercomplexes undergo a remodeling caused by their complete or partial dissociation into individual proteins or small protein subcomplexes.

**Figure 6:**
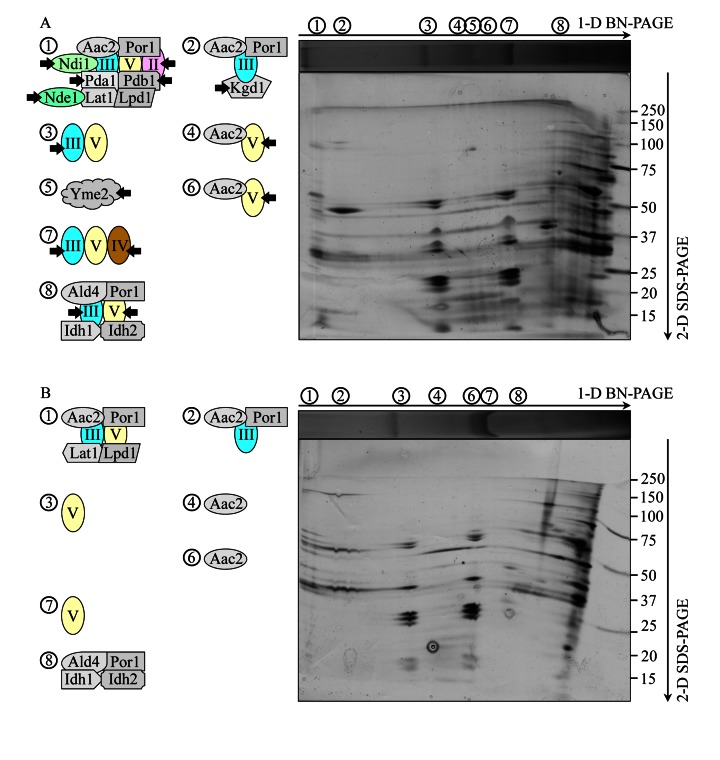
Under CR conditions, the *atg32Δ* mutation eliminates the non-respiratory protein supercomplex 5 in the IMM and alters compositions of other protein supercomplexes recovered by 1-D BN-PAGE WT and *atg32Δ* strains were cultured in the nutrient-rich YP medium initially containing 0.2% glucose. Mitochondria were purified from WT and *atg32Δ* cells recovered on day 4 of culturing. Digitonin-solubilized protein supercomplexes from the inner membrane of mitochondria purified from WT (**A**) or *atg32Δ* (**B**) cells were separated by first-dimension blue native PAGE (1-D BN-PAGE) and then resolved by second-dimension tricine-SDS-PAGE (2-D SDS-PAGE). Following silver staining, the separated by 2-D SDS-PAGE proteins were identified by mass spectrometry. Arrows in **A** mark the non-respiratory protein supercomplex 5 lacking in the IMM of *atg32Δ* cells as well as individual proteins or respiratory protein complexes dissociated from other protein supercomplexes in the IMM of these mutant cells.

To define the composition of each of the protein supercomplexes recovered by 1-D BN-PAGE (Figure 5A), we resolved their individual protein components by second-dimension tricine-SDS-PAGE (2-D SDS-PAGE), visualized these components by silver staining and identified them by mass spectrometry. We found that, in addition to one or more kinds of individual respiratory complexes, many protein supercomplexes in the IMM of WT cells contain one or more proteins that have not been traditionally viewed as components associated (even transiently) with the mitochondrial respiratory chain. These novel protein components of respiratory protein supercomplexes in the IMM of WT cells include: (i) the ADP-ATP carrier protein Aac2p, a component of supercomplexes 1, 2, 4 and 6; (ii) the mitochondrial porin Por1p, a component of supercomplexes 1, 2 and 8; (iii) the Pda1p, Pdb1p, Lat1p and Lpd1p subunits of the mitochondrial pyruvate dehydrogenase complex, components of the supercomplex 1; (iv) the mitochondrial external NADH dehydrogenase Nde1p, a component of the supercomplex 1; (v) the Kgd1p subunit of the mitochondrial alpha-ketoglutarate dehydrogenase complex, a component of the supercomplex 2; (vi) the Ald4p isoform of acetaldehyde dehydrogenase, a component of the supercomplex 8; and (vii) the Idh1p and Idh2p isoforms of isocitrate dehydrogenase, a component of the supercomplex 8 (Figure 6A; Table S1). Moreover, the protein supercomplex 5 in the IMM of WT cells is a non-respiratory supercomplex composed of many molecules of Yme2p (Figure 6A; Table S1), an integral IMM protein with a role in maintaining mitochondrial nucleoid structure and number. It should be stressed that the *atg32Δ* mutation not only eliminates this non-respiratory supercomplex 5 but also causes a dissociation of several proteins or protein complexes from supercomplexes 1, 2, 3, 4, 6, 7 and 8. Some of these dissociated in *atg32Δ* cells proteins or protein complexes are known as components of the mitochondrial respiratory chain, including (i) Ndi1p, a dissociated from the supercomplex 1 oxidoreductase that (akin to the mitochondrial respiratory complex I in higher eukaryotes) transfers electrons from NADH to ubiquinone in the respiratory chain but (unlike respiratory complex I in higher eukaryotes) does not pump protons; (ii) the respiratory complex III, which dissociates from supercomplexes 3, 7 and 8; (iii) the respiratory complex IV, which dissociates from the supercomplex 7; and (iv) the respiratory complex V, which dissociates from supercomplexes 4, 6 and 8 (Figures 6A and 6B; Table S1). Other of the dissociated in *atg32Δ* cells proteins or protein complexes have not been traditionally viewed as components associated (even transiently) with the mitochondrial respiratory chain; they include (i) dissociated from the supercomplex 1 subunits Pda1p, Pdb1p, Lat1p and Lpd1p of the mitochondrial pyruvate dehydrogenase complex; (ii) a dissociated from the supercomplex 1 mitochondrial external NADH dehydrogenase Nde1p; and (iii) a dissociated from the supercomplex 2 subunit Kgd1p of the mitochondrial alpha-ketoglutarate dehydrogenase complex (Figures 6A and 6B; Table S1).

In sum, these data suggest that under CR conditions macromitophagy is required to sustain a population of mitochondria whose inner membrane exhibits abundant protein supercomplexes, each composed of a distinct set of respiratory and non-respiratory protein complexes. One could therefore foresee that in chronologically aging yeast limited in calorie supply macromitophagy selectively eliminates mitochondria whose inner membrane (i) has reduced levels of respiratory and non-respiratory protein supercomplexes 2, 3, 4 and 5 due to their complete dissociation into individual proteins or small protein subcomplexes; (ii) accumulates respiratory and non-respiratory protein supercomplexes 1, 3, 4, 6, 7 and 8 that lack one or more respiratory complexes; (iii) amasses respiratory and non-respiratory protein supercomplexes 1, 2 and 8 that lack proteins not traditionally viewed as components associated with the mitochondrial respiratory chain; and/or (iv) has diminished level of the non-respiratory protein supercomplex 5 composed of many molecules of Yme2p, an integral IMM protein involved in maintaining mitochondrial nucleoid structure and number.

### Macromitophagy preserves a population of mitochondria that efficiently couple electron transport to ATP synthesis and exhibit high and balanced activities of all five oxidative phosphorylation (OXPHOS) complexes

We sought to investigate if the observed in macromitophagy-deficient *atg32Δ* cells reduced abundance and altered composition of the major respiratory and non-respiratory protein supercomplexes (Figures 5 and 6) have an impact on the functional state of the electron transport and OXPHOS system in the IMM of these prematurely aging mutant cells.

To attain this objective, we used polarography of mitochondria purified from WT and *atg32Δ* cells for measuring the following three kinds of respiratory rate: (i) the rate of state III (also called ADP-stimulated state) respiration [36, 37], which was assessed immediately following addition of 50 μM ADP to mitochondria supplemented with a respiratory substrate; (ii) the rate of state IV (also known as resting, basal or ADP-independent state) respiration [37, 38], which was monitored after the exogenously added ADP was entirely converted to ATP; and (iii) the rate of uncoupled (UC) respiration [37, 39], which was determined after addition of the uncoupler carbonyl cyanide m-chlorophenylhydrazone (CCCP) to mitochondria incubated with a respiratory substrate in the absence of exogenous ADP. In these polarographic assays, the rates of state III, state IV and UC respiration were measured using NADH as a respiratory substrate of external NADH:quinone oxidoreductase or succinate as a respiratory substrate of complex II.

**Figure 7:**
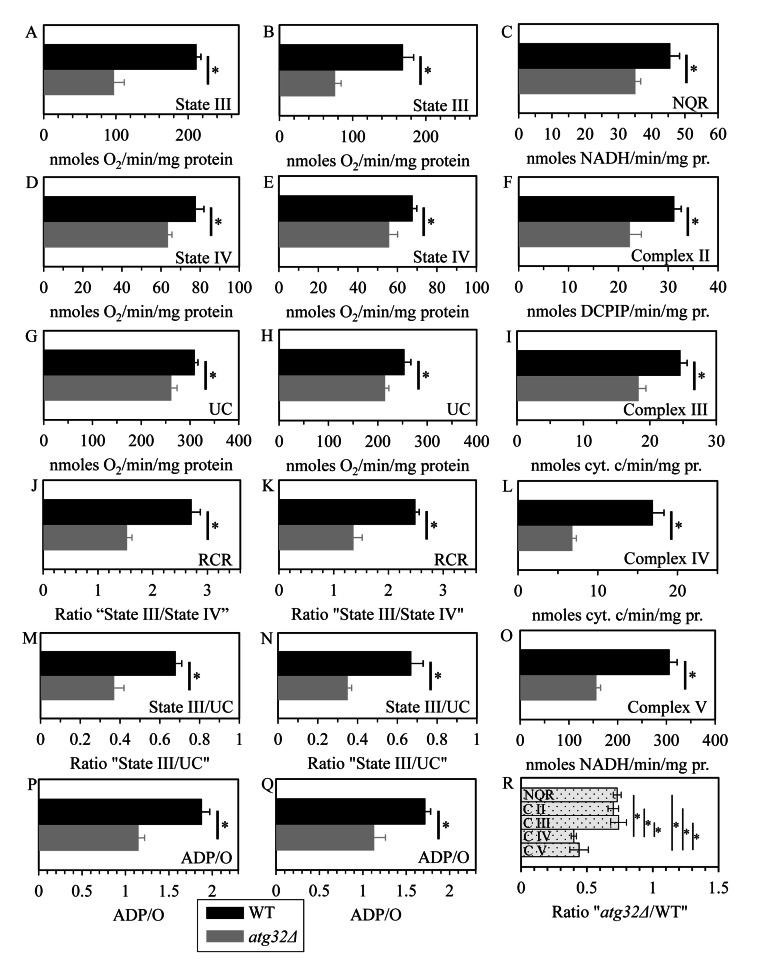
Under CR conditions, the *atg32Δ* mutation reduces capacity of the mitochondrial ETC, lowers the efficacy of coupling between ADP phosphorylation and electron transport, compromises the integrity of the IMM, and disproportionally decreases activities of all OXPHOS enzymes WT and *atg32Δ* strains were cultured in the nutrient-rich YP medium initially containing 0.2% glucose. Mitochondria were purified from WT and *atg32Δ* cells recovered on day 4 of culturing. The rates of state III (**A** and **B**), state IV (**D** and **E**) and UC (**G** and **H**) respiration were measured using NADH (**A**, **D** and **G**) or succinate (**B**, **E** and **H**) as a respiratory substrate. The ratios of state III rate/state IV rate (RCR; **J** and **K**), state III rate/UC rate (**M** and **N**) and ADP/O (**P** and **Q**) were determined using NADH (**J**, **M** and **P**) or succinate (**K**, **N** and **Q**) as a respiratory substrate. Enzymatic activities of the OXPHOS enzymes NADH:decylubiquinone oxidoreductase (NQR; **C**), succinate:decylubiquinone-2,6-dichlorophenolindo-phenol oxidoreductase (complex II) (**F**), ubiquinol:cytochrome *c* oxidoreductase (complex III) (**I**), cytochrome *c* oxidase (complex IV) (**L**) and F_1_F_0_-ATP synthase (complex V) (**O**) were assessed. For each of these OXPHOS enzymes, the ratio “activity in mitochondria of *atg32Δ* strain/activity in mitochondria of WT strain” was calculated (**R**).

We found that the *atg32Δ* mutation significantly reduces the rates of state IV (Figures 7D and 7E) and UC (Figures 7G and 7H) respiration in the presence of NADH or succinate as a respiratory substrate. Both these respiratory rates are known to reflect the capacity of the electron transport chain (ETC) in the IMM [36, 37]. Thus, in cells grown under CR conditions on 0.2% glucose macromitophagy is required to preserve a population of mitochondria that exhibit high capacity of electron transport along the respiratory chain.

We also found that the *atg32Δ* mutation considerably decreases (i) the rate of state III respiration (Figures 7A and 7B); (ii) the state III rate/state IV rate ratio (known as the respiratory control ratio, RCR) (Figures 7J and 7K); (iii) the state III rate/UC rate ratio (Figures 7M and 7N); and (iv) the ADP/O ratio (*i.e.*, the number ATP molecules synthesized per oxygen atom reduced) (Figures 7P and 7Q). All these parameters are known to reflect the extent of the functional and physical integrity of the IMM; the ADP/O ratio is also a direct measure of the efficacy of coupling between ADP phosphorylation and electron transport along the respiratory chain [36, 37, 39]. We therefore concluded that in yeast cells limited in calorie supply macromitophagy is obligatory for sustaining a population of mitochondria that (i) preserve the functional and physical integrity of the IMM; and (ii) efficiently couple electron transport to ATP synthesis.

To get insight into the effect of the observed in macromitophagy-deficient *atg32Δ* cells reduced abundance and altered composition of the major respiratory and non-respiratory protein supercomplexes on the functional state of each of the five OXPHOS protein complexes, we measured their enzymatic activities in mitochondria purified from WT and *atg32Δ* cells grown under CR conditions on 0.2% glucose.

As we found, the *atg32Δ* mutation causes a significant reduction in enzymatic activities of NADH: decylubi- quinone oxidoreductase (NQR; an impaired in proton pumping across the IMM analog of the respiratory complex I in higher eukaryotes), succinate:decylubiquinone-2,6-dichlorophenolindo-phenol oxidoreductase (the respiratory complex II), ubiquinol:cytochrome c oxidoreductase (the respiratory complex III), cytochrome c oxidase (the respiratory complex IV) and F1F0-ATP synthase (the respiratory complex V) (Figures 7C, 7F, 7I, 7L and 7O). Noteworthy, the extent of such reduction for the respiratory complexes IV and V substantially exceeded that for NQR as well as for the respiratory complexes II and III (Figure 7R). Hence, in yeast cells limited in calorie supply macromitophagy is needed for maintaining a population of mitochondria that display high and balanced activities of all five OXPHOS complexes.

Altogether, our polarographic and enzymatic assays in mitochondria purified from WT and *atg32Δ* cells suggest that in chronologically aging yeast under CR conditions macromitophagy may selectively eliminate mitochondria whose inner membrane exhibits (i) a compromised functional and physical integrity of the IMM; (ii) disproportionally reduced activities of the five OXPHOS complexes; (iii) a low capacity of electron transport along the respiratory chain; and (iv) a reduced efficacy of coupling between ADP phosphorylation and electron transport.

### Macromitophagy sustains a population of mitochondria that generate low levels of ROS and exhibit only a minor oxidative damage to mitochondrial macromolecules

**Figure 8:**
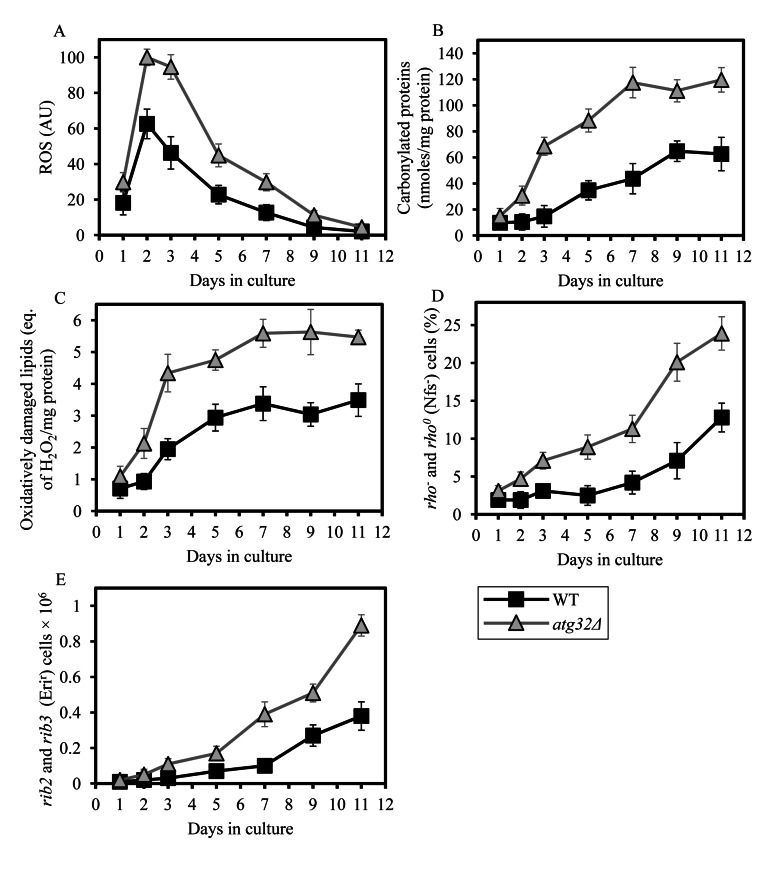
Under CR conditions, the *atg32Δ* mutation increases the level of mitochondrially produced ROS, elevates the extent of oxidative damage to mitochondrial proteins and membrane lipids, and rises the frequencies of mutations in mtDNA WT and *atg32Δ* strains were cultured in the nutrient-rich YP medium initially containing 0.2% glucose. (**A**) The dynamics of age-dependent changes in the intracellular levels of ROS during chronological aging of yeast. ROS were visualized using dihydrorhodamine 123 (DHR). At least 1,000 cells were used for quantitation of DHR staining for each of 4 independent experiments. Data are presented as mean ± SEM. (**B** and **C**) Carbonylated proteins (**B**) and oxidatively damaged membrane lipids (**C**) in purified mitochondria were determined as described in Methods. Data are presented as mean ± SEM (n = 2-3). (**D**) The percentage of respiratory-deficient cells unable to grow in medium containing 3% glycerol because they carried large mtDNA deletions (*rho^−^*) or lacked mtDNA (*rho°*). Data are presented as mean ± SEM (n = 5). (**E**) The frequencies of *rib2* and *rib3* point mutations in mtDNA caused resistance to erythromycin. Data are presented as mean ± SEM (n = 4).

A body of evidence supports the view that the multifactorial functional state of the mitochondrial ETC and OXPHOS system in the inner membrane of yeast mitochondria (rather than just the total number of electrons transported along the respiratory chain) determines the rate of mitochondrial ROS production, thereby modulating the extent of oxidative cellular damage and other vital processes defining yeast longevity [40-44]. It is well established that the functional state of the mitochondrial ETC and coupled OXPHOS system in yeast is influenced in part by the rate of mitochondrial respiration, capacity of electron flow through the respiratory chain, number and activity of individual OXPHOS enzymes, and relative levels of the five OXPHOS complexes [40-42, 44-46]. We demonstrated that in yeast limited in calorie supply the *atg32Δ*-dependent mutational block of macromitophagy reduces mitochondrial respiration, decreases capacity of the mitochondrial ETC, lowers the efficacy of coupling between ADP phosphorylation and electron transport, and disproportionally lessens activities of all OXPHOS enzymes (Figures 4A and 7). This observation prompted us to investigate if the longevity-shortening *atg32Δ* mutation alters the age-related dynamics of changes in mitochondrially generated ROS and/or impacts the extent of oxidative damage to mitochondrial macromolecules. We found that in chronologically aging yeast cultured under CR on 0.2% glucose and recovered at different periods of CLS, the *atg32Δ* mutation elevates the level of mitochondrially produced ROS (Figure 8A). Furthermore, our comparative analysis of mitochondria purified from WT and *atg32Δ* mutant cells limited in calorie supply revealed that this mutation increases the extent of oxidative carbonylation of mitochondrial proteins (Figures 8B and S1) and elevates the levels of oxidatively damaged mitochondrial membrane lipids (Figure 8C). We also observed that *atg32Δ* mutant cells grown under CR on 0.2% glucose exhibited augmented, as compared to WT cells, frequencies of deletion (*rho^−^* and *rho^0^*) and point (*rib2* and *rib3*) mutations in mitochondrial DNA (mtDNA) (Figures 8D and 8E) - likely due to an elevated extent of oxidative damage to mtDNA in these mutant cells.

Taken together, these findings suggest that under CR conditions macromitophagy is required to sustain a population of mitochondria that generate low levels of ROS and exhibit only a minor oxidative damage to mitochondrial proteins, membrane lipids and DNA. Thus, it is conceivable that in chronologically aging yeast limited in calorie supply macromitophagy selectively eliminates mitochondria producing excessively high concentrations of ROS and accumulating elevated levels of macromolecules oxidatively damaged due to their exposure to mitochondrial ROS. One could predict that the inability of macromitophagy-deficient *atg32Δ* cells to eliminate these oxidatively impaired (and therefore dysfunctional) mitochondria may result in their progressive accumulation with age, thereby shortening yeast longevity.

### Macromitophagy is required for maintaining the homeostasis of membrane lipids in mitochondria, the ER and the PM

Mitochondria are known to be the only site within a yeast cell where cardiolipin (CL), the signature glycerophospholipid of these organelles, is synthesized [25, 26]. Furthermore, yeast mitochondria house the synthesis of the glycerophospholipid phosphatidy-lethanolamine (PE), minor quantities of which are also formed in the Golgi apparatus [26]. It should be emphasized that both CL and PE are synthesized in the the inner membrane of yeast mitochondria from their precursor lipid species formed exclusively in the ER; cytidine diphosphate-diacylglycerol (CDP-DAG) serves as a precursor for CL, whereas the glycerophospholipid phosphatidylserine (PS) is a precursor for PE (Figure S2) [25]. Thus, the confined to mitochondria synthesis of CL and PE in yeast cells relies on a delivery of their CDP-DAG and PS precursors from the ER to the IMM [21, 25, 47]. Moreover, the mitochondrially synthesized PE serves as a precursor for the glycerophospholipid phosphatidylcholine (PC), which is synthesized only in the ER (Figure S2) [26, 27]. Hence, following its synthesis in the ER, PC must be transported to mitochondria incapable of synthesizing this essential glycerophospholipid constituent of both mitochondrial membranes (Figure S2) [25, 47]. In addition, the glycerophospholipids phosphatidic acid (PA) and phosphatedylinositol (PI) are synthesized in the ER but not in mitochondria of yeast cells; therefore, these two lipid species playing essential roles in mitochondrial membranes need to be translocated to these membranes from the site of their synthesis in the ER (Figure S2) [25, 26]. Altogether, these findings imply that the normal functioning of yeast mitochondria requires a bidirectional lipid exchange between the mitochondrial and ER membranes. Such an exchange occurs via zones of close apposition, known as membrane contact sites or junctions, between the outer mitochondrial membrane and the mitochondria-associated membrane (MAM) domain of the ER; from 80 to 110 of these mitochondria-ER junctions are believed to exist per yeast cell (Figure S2) [20, 21, 27]. Of note, following their synthesis within the ER and inner mitochondrial membranes, lipids can be translocated to the PM; as many as 1,100 PM-ER junctions per yeast cell are involved in lipid transport from the PM-associated membrane (PAM) domain of the ER to the PM (Figure S2) [21-24].

Considering such intricate integration of mitochondria into a network governing lipid dynamics not only within these organelles but also within the ER and the PM, we hypothesized that the observed inability of prematurely aging under CR conditions *atg32Δ* cells to eliminate morphologically distinct, dysfunctional and oxidatively damaged mitochondria may compromise lipid metabolism and movement within the entire network. One could therefore envisage that the *atg32Δ*-dependent mutational block of macromitophagy may alter membrane lipid composition not only in mitochondria but also in the ER and the PM.

**Figure 9:**
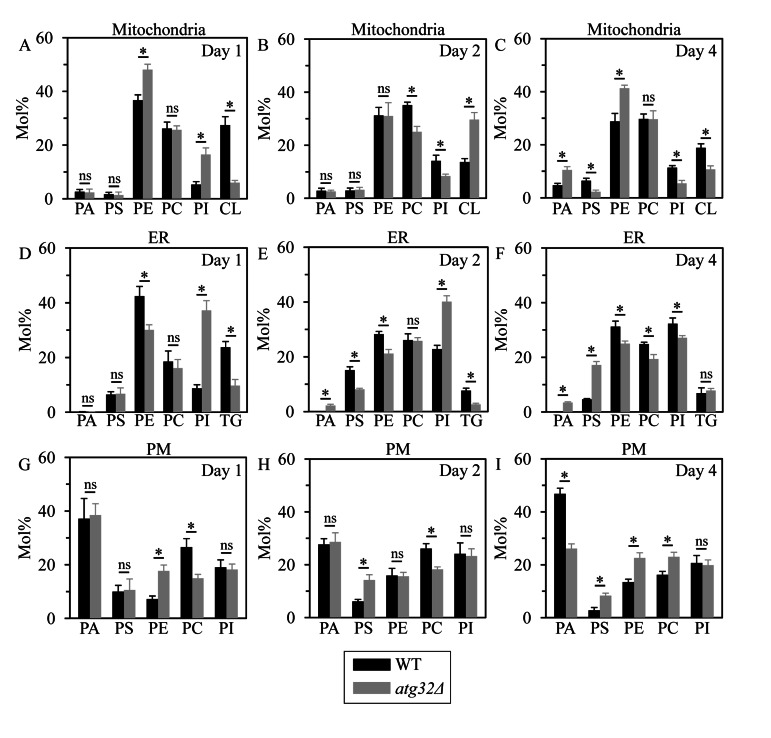
Under CR conditions, the *atg32Δ* mutation alters levels of several membrane lipid species in mitochondria, the ER and the PM WT and *atg32Δ* strains were cultured in the nutrient-rich YP medium initially containing 0.2% glucose. Mitochondria, the ER and the PM were purified from WT and *atg32Δ* cells recovered on day 1, 2 or 4 of culturing. Following extraction of membrane lipids from purified mitochondria, ER and PM, various lipid species were identified and quantitated by mass spectrometry as described in Methods. Data are presented as means ± SEM (n = 3; *p < 0.01; ns, not significant).

To test the validity of our hypothesis, we used mass spectrometry to compare the membrane lipidomes of mitochondria, ER and PM purified from WT and *atg32Δ* cells that were cultured under CR on 0.2% glucose and recovered on day 1, 2 or 4 of culturing. We found that (i) the *atg32Δ* mutation considerably alters levels of several membrane lipid species in mitochondria, ER and PM; and (ii) the effect of *atg32Δ* on the membrane lipidomes of mitochondria, the ER and the PM is different in cells recovered on day 1, 2 or 4 of culturing - and, thus, is age-related (Figure 9).

Indeed, in cells recovered at logarithmic growth phase on day 1, the *atg32Δ* mutation (i) increased the levels of PE (a glycerophospholipid delivered to the ER after its synthesis in the IMM) in mitochondria and the PM, and reduced its level in the ER; (ii) lowered the level of CL (a mitochondrially synthesized and accumulated glycerophospholipid) in mitochondria; (iii) decreased the level of PC (a glycerophospholipid synthesized in the ER membrane and then delivered to other membranes) in the PM, but did not alter its abundance in mitochondria or the ER; (iv) elevated the levels of PI (a glycerophospholipid synthesized in the ER membrane and then delivered to other membranes) in mitochondria and the ER, but did not alter its abundance in the PM; (v) lowered the level of TG (neutral lipid species synthesized in the ER but not in mitochondria or the PM) in the ER; and (vi) did not affect the levels of PA and PS (two glycerophospholipids synthesized in the ER membrane and then delivered to other membranes) in mitochondria, the ER and the PM (Figures 9A, 9D and 9G).

Furthermore, in cells recovered at diauxic growth phase on day 2, the *atg32Δ* mutation (i) reduced the level of PE in the ER membrane but not in mitochondria or the PM; (ii) elevated the level of CL in mitochondria; (iii) decreased the levels of PC in mitochondria and the PM, but did not alter its abundance in the ER; (iv) reduced the level of PI in mitochondria and elevated the level of this glycerophospholipid in the ER, but did not alter its abundance in the PM; (v) lowered the level of TG in the ER; (vi) increased the level of PA in the ER membrane, but did not alter its abundance in mitochondria or the PM; and (vii) reduced the level of PS in the ER and elevated the level of this glycerophospholipid in the PM, but did not alter its abundance in mitochondria (Figures 9B, 9E and 9H).

Moreover, in cells recovered at post-diauxic growth phase on day 4, the *atg32Δ* mutation (i) elevated the levels of PE in mitochondria and the PM, and reduced its abundance in the ER; (ii) decreased the level of CL in mitochondria; (iii) reduced the level of PC in the ER and increased the level of this glycerophospholipid in the PM, but did not alter its abundance in mitochondria; (iv) lowered the levels of PI in mitochondria and the ER, but did not alter its abundance in the PM; (v) did not alter the level of TG in the ER; (vi) elevated the levels of PA in mitochondria and the ER, and reduced its abundance in the PM; and (vii) decreased the level of PS in mitochondria, but increased the levels of this glycerophospholipid in the ER and the PM (Figures 9C, 9F and 9I).

Altogether, these findings imply that under CR conditions macromitophagy is essential for sustaining the homeostasis of membrane lipids in mitochondria, the ER and the PM. It is plausible that the observed in macromitophagy-deficient *atg32Δ* cells age-related changes in the membrane lipidomes of mitochondria, the ER and the PM may shorten yeast longevity by establishing a pro-aging cellular pattern.

## DISCUSSION

CR is a diet in which only calorie availability is limited but the supply of amino acids, nucleotides, vitamins and other nutrients is not compromised [48]. This dietary intervention is known for its robust longevity-extending and health-improving effects across species [48-52], although recent findings in mice and primates initiated an intense debate over some of the beneficial effects attributed to CR [53, 54]. In this study, we provide the first evidence that selective macroautophagic mitochondrial removal plays a pivotal role in longevity extension by a CR diet in chronologically aging yeast; such a diet was implemented by culturing yeast cells in a nutrient-rich medium initially containing low (0.2%) concentration of glucose, a fermentable carbon source. It should be emphasized that under these longevity-extending CR conditions yeast cells are not starving but undergo an extensive remodeling of their metabolism in order to match the level of ATP produced under longevity-shortening non-CR conditions [18, 55]. Moreover, our study also reveals that in chronologically aging yeast limited in calorie supply macromitophagy is essential for longevity extension by LCA. This bile acid is a potent anti-aging intervention previously shown to act in synergy with CR to enable a significant further extension of yeast lifespan under CR conditions by modulating so-called “housekeeping” longevity pathways [19, 56]. In sum, these findings imply that macromitophagy is a longevity assurance process that in chronologically aging yeast underlies the synergistic beneficial effects of anti-aging dietary and pharmacological interventions (*i.e.*, CR and LCA) on lifespan.

Our data suggest that macromitophagy can maintain survival of chronologically aging yeast limited in calorie supply by controlling a compendium of vital cellular processes known for their essential roles in defining longevity, as outlined below.

First, the observed in short-lived *atg32Δ* cells significant reduction in the level of ATP implies that macromitophagy can selectively eliminate mitochondria incapable of producing this universal energy source in quantities sufficient to drive pro-longevity cellular processes. The diminished ATP production by mitochondria accumulating in macromitophagy-deficient *atg32Δ* cells is likely due to their demonstrated inability to sustain a population of mitochondria whose inner membrane exhibits abundant protein supercomplexes, each composed of a distinct set of respiratory and non-respiratory protein complexes. It is conceivable that such an inability causes the observed in *atg32Δ* cells build-up of mitochondria that exhibit (i) a decreased respiration rate; (ii) a reduced electro-chemical potential across the IMM; (iii) an impaired functional and physical integrity of the IMM; (iv) disproportionally lowered activities of all five OXPHOS complexes; (v) a reduced capacity of electron transport along the respiratory chain; and (vi) a low efficacy of coupling between ADP phosphorylation and electron transport. The build-up of these dysfunctional mitochondria can be progressively accelerated by the demonstrated in prematurely aging *atg32Δ* cells age-related accumulation of mitochondria that (i) generate high levels of ROS; (ii) display a major oxidative damage to mitochondrial proteins and lipids; and (iii) exhibit increased frequencies of mtDNA mutations likely caused by excessive levels of mitochondrial ROS.

Second, the observed in prematurely aging *atg32Δ* cells substantial rise of ROS in mitochondria, the major cellular site of ROS formation as by-products of respiration [57-59], implies that macromitophagy can selectively eliminate mitochondria incapable of maintaining intracellular ROS concentration below a toxic threshold. Because both mitochondrial membranes are permeable to the hydrogen peroxide form of ROS [57-59], the elevated ROS production by mitochondria accumulating in macromitophagy-deficient*atg32Δ* cells is likely to accelerate yeast aging by causing an excessive oxidative damage to extramitochondrial cellular macromolecules. To test the validity of this hypothesis, we are currently investigating an impact of the *atg32Δ* mutation on the levels of oxidatively damaged proteins, lipids and nucleic acids that reside outside mitochondria.

Third, the observed in short-lived *atg32Δ* cells massive fragmentation of the elaborate mitochondrial network, reduction of the electrochemical potential across the IMM, release of cytochrome *c* from the intermediate space of mitochondria into the cytosol and elevated level of ROS imply that macromitophagy can selectively eliminate mitochondria that exhibit MOMP and the so-called mitochondrial permeability transition (MPT). MOMP is known to trigger apoptotic cell death, whereas MPT promotes necrotic cell death [29, 60]. One could anticipate that the demonstrated in this study early loss of viability by prematurely aging *atg32Δ* cells may be due to their accelerated apoptotic or necrotic death controlled by mitochondria. It should be emphasized that both these cell death modalities have been shown to define yeast longevity [18, 19, 56, 61, 62]. It is conceivable therefore that macromitophagy can maintain survival of chronologically aging yeast limited in calorie supply by eliminating only mitochondria capable of triggering an age-related form of apoptotic or necrotic cell death initiated by MOMP and/or MPT. A challenge for the future will be to use chronologically aging yeast under CR conditions as a valuable model for elucidating an impact of the *atg32Δ* mutation on the spatiotemporal dynamics of various hallmark events characteristic of apoptotic and necrotic modes of cell death.

Fourth, the observed in *atg32Δ* cells grown under CR conditions substantial reduction of both mitochondrial respiration and the electrochemical potential across the IMM has been shown to stimulate activity of the cAMP-dependent protein kinase A (PKA) in WT yeast cells cultured under conditions of amino acid starvation [63]. PKA is known to govern several longevity-defining processes in chronologically aging yeast [64]. If activated, this nutrient-sensory protein kinase (i) inhibits a housekeeping, anti-aging process of non-selective macroautophagy [63, 65, 66]; (ii) activates a pro-aging process of protein synthesis in the cytosol [67]; and (iii) inhibits nuclear import of Msn2p and Msn4p, thus turning off an anti-aging transcriptional program driven by these two stress response transcriptional activators [67, 68]. One could predict that macromitophagy can maintain survival of chronologically aging yeast limited in calorie supply by eliminating mitochondria exhibiting a decreased respiration rate and a reduced electrochemical potential across the IMM, thereby attenuating PKA signalling to establish a pro-longevity cellular pattern. It would be important to test the validity of this hypothesis in the near future by assessing the effects of the *atg32Δ* mutation on non-selective macroautophagy, protein synthesis in the cytosol, nuclear import of Msn2p and Msn4p, and transcriptional pattern in yeast cultured under CR conditions.

**Figure 10:**
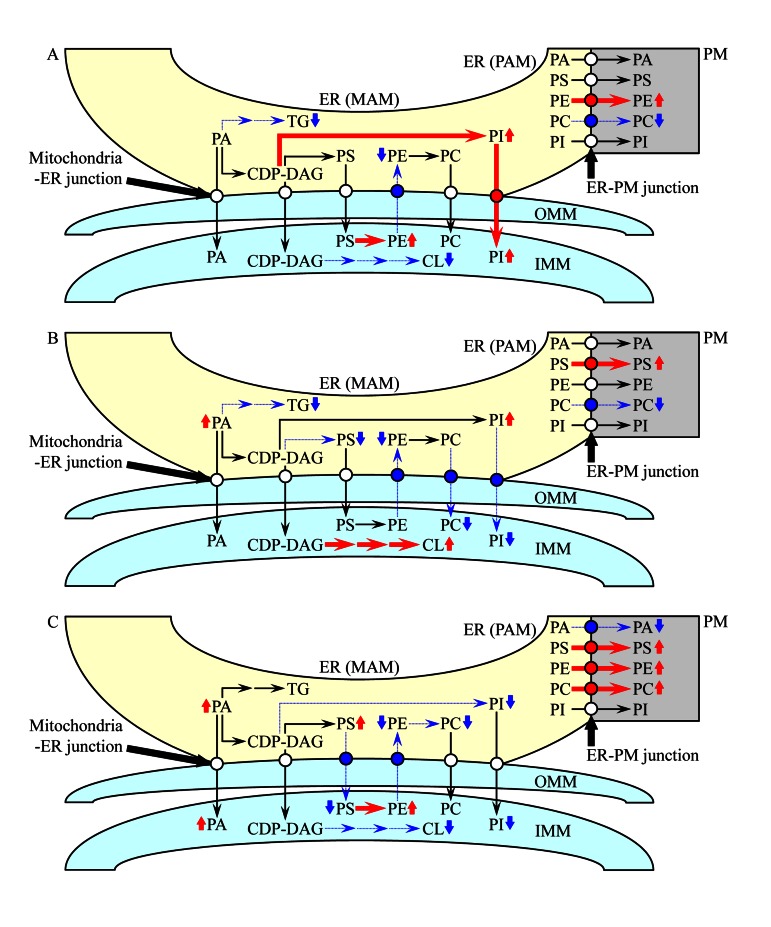
A working model for a mechanism that in *atg32Δ* cells underlies the spatiotemporal dynamics of age-related changes in lipid synthesis in the ER and mitochondria as well as in lipid transport via mitochondria-ER (MAM) and PM-ER (PAM) junctions During logarithmic (**A**), diauxic (**B**) and post-diauxic (**C**) phases of growth under CR conditions, a remodelling of lipid synthesis and transport in *atg32Δ* cells alters the membrane lipidomes of mitochondria, the ER and the PM. From the data of lipidomic analysis, we inferred an outline of lipid synthesis and transport processes that were activated (red arrows) or inhibited (blue arrows) by the *atg32Δ*-dependent mutational block of macromitophagy; the thickness of these arrows correlates with the rates of the processes taking place in *atg32Δ* cells. Arrows next to the names of lipid species denote those of them whose concentrations are elevated (red arrows) or reduced (blue arrows) in *atg32Δ* cells. See text for details.

Fifth, the observed in *atg32Δ* cells age-related changes in the membrane lipidomes of mitochondria, the ER and the PM imply that macromitophagy selectively eliminates mitochondria exhibiting an imbalance of their membrane lipidome, perhaps due to progressing with age alterations in the synthesis, stability and/or oxidative state of some lipid species. Because mitochondria are convolutedly integrated into a network governing lipid dynamics not only within these organelles but also within the ER and the PM (Figure S2), it is reasonable to assume that the inability of macromitophagy-deficient *atg32Δ* cells to eliminate mitochondria exhibiting such an age-related imbalance of their membrane lipidome (i) can also compromise lipid metabolism and transport in the ER and the PM; and (ii) is therefore responsible for the observed in *atg32Δ* cells changes in the membrane lipidomes of mitochondria, the ER and the PM. Our findings suggest a working model for a mechanism that in macro-mitophagy-deficient*atg32Δ* cells underlies the spatiotemporal dynamics of age-related alterations in lipid synthesis in the ER and mitochondria as well as in lipid transport via mitochondria-ER (MAM) and PM-ER (PAM) junctions (Figure 10). During logarithmic growth phase, a remodelling of lipid synthesis and transport in *atg32Δ* cells leads to the following changes in the membrane lipidomes of mitochondria, the ER and the PM: (i) an activated conversion of PS into PE in the IMM and a simultaneous inhibition of PE transport from mitochondria to the MAM domain of the ER via mitochondria-ER (MAM) junctions elevate PE concentration in mitochondria and reduce its concentration in the ER; (ii) an activated synthesis of PI from CDP-DAG in the MAM domain of the ER, along with an activation of PI transport from this ER domain to mitochondria via mitochondria-ER (MAM) junctions, rise PI concentration in both mitochondria and the ER; (iii) an inhibition of TG synthesis from PA in the MAM domain of the ER reduces TG concentration in this organelle; (iv) an inhibition of CL synthesis from CDP-DAG in the IMM is responsible for the observed decline of CL concentration in mitochondria; (v) an activated transport of PE from the PAM domain of the ER to the PM via PM-ER (PAM) junctions increases PE concentration in the PM; and (vi) an inhibition of PC transport from the PAM domain of the ER to the PM via PM-ER (PAM) junctions reduces PC concentration in the PM (Figure 10A). During diauxic growth phase, *atg32Δ* cells undergo a remodelling of lipid synthesis and transport causing the following alterations to membrane lipid concentrations in mitochondria, the ER and the PM: (i) an inhibited conversion of CDP-DAG into PS in the MAM domain of the ER and an inhibition of PE transport from mitochondria to this ER domain via mitochondria-ER (MAM) junctions reduce the concentrations of both PS and PE in the ER; (ii) an inhibition of PC transport from the MAM domain of the ER to mitochondria via mitochondria-ER (MAM) junctions lowers the concentration of this glycerol-phospholipid in mitochondria; (iii) an inhibited transport of PI from the MAM domain of the ER to mitochondria via mitochondria-ER (MAM) junctions reduces PI concentration in mitochondria and rises its level in the ER; (iv) an activation of CL synthesis from CDP-DAG in the IMM leads to the observed increase of CL concentration in mitochondria; (v) an inhibited conversion of PA into TG in the MAM domain of the ER is responsible for the observed rise of PA and decline of TG concentrations in this organelle; (vi) an activated transport of PS from the PAM domain of the ER to the PM via PM-ER (PAM) junctions increases its concentration in the PM; and (vi) an inhibition of PC transport from the PAM domain of the ER to the PM via PM-ER (PAM) junctions reduces PC level in the PM (Figure 10B). During post-diauxic growth phase, a remodelling of lipid synthesis and transport in *atg32Δ* cells alters the membrane lipidomes of mitochondria, the ER and the PM as follows: (i) an inhibition of PS transport from the MAM domain of the ER to mitochondria and a simultaneous inhibition of PE transport in the opposite direction via mitochondria-ER (MAM) junctions, along with an activated conversion of PS into PE in the IMM, not only rise the concentrations of PS in the ER and PE in mitochondria but also reduce the levels of PE in the ER and PS in mitochondria; (ii) an inhibited synthesis of PC from PE in the MAM domain of the ER lowers PC concentration in this organelle; (iii) an inhibition of the conversion of CDP-DAG into PI in the MAM domain of the ER reduces PI levels both in the ER and in mitochondria; (iv) an inhibited synthesis of CL from CDP-DAG in the IMM lowers CL concentration in mitochondria; (v) an activated transport of PS, PE and PC from the PAM domain of the ER to the PM via PM-ER (PAM) junctions increases their concentrations in the PM; and (vi) an inhibition of PA transport from the PAM domain of the ER to the PM via PM-ER (PAM) junctions not only reduces PA level in the PM but may also be responsible for the observed rise of PA concentrations in the ER and mitochondria (Figure 10C).

It should be emphasized that the spatiotemporal dynamics of lipid synthesis, deposition and degradation has been shown to play an essential role in longevity regulation in evolutionarily distant organisms, including yeast [12, 18, 55, 69]. One could therefore envisage that progressing with age substantial changes in the membrane lipidomes of mitochondria, the ER and the PM, as those seen in prematurely aging *atg32Δ* cells, may cause the establishment of a pro-aging cellular pattern and may ultimately shorten yeast longevity. In the future, it would be important to identify longevity-defining cellular processes that are responsive to the observed in *atg32Δ* cells alterations in the repertoires of membrane lipids constituting mitochondria, the ER and the PM. These cellular processes may include (i) a mitochondria-controlled, age-related mode of apoptotic cell death known to be modulated in response to changes in CL concentration within the IMM [70, 71]; and (ii) a non-selective pathway of macroautophagy shown to be responsive to certain alterations in lipid metabolism within and lipid exchange between several cellular membranes [72-74].

Another challenge for the future will be to establish which of the numerous morphological and physiological features exhibited by dysfunctional mitochondria that amass in *atg32Δ* cells direct only a population of dysfunctional mitochondria for selective macro-mitophagic destruction in WT cells limited in calorie supply. It would be also important to investigate if in WT cells grown under CR only a pool of Atg32p confined to dysfunctional mitochondria is subjected to phosphorylation, as it has been seen in yeast undergoing macromitophagy triggered in response to nitrogen starvation [75, 76]. Moreover, it remains to be seen which of the mitophagy-linked proteins other than Atg32p (a number of such proteins have been identified in the genome-wide screens as products of genes essential for mitophagy [16, 17]) are involved in macromitophagy in chronologically aging WT cells limited in calorie supply.

## METHODS

### Yeast strains, media and growth conditions

The wild-type strain *Saccharomyces cerevisiae* BY4742 (*MAT*α *his3Δ1 leu2Δ0 lys2Δ0 ura3Δ0*) as well as the single-gene-deletion mutant strains *atg32Δ* (*MAT*α *his3Δ1 leu2Δ0 lys2Δ0 ura3Δ0 atg32Δ::kanMX4*) and *atg36Δ* (*MAT*α *his3Δ1 leu2Δ0 lys2Δ0 ura3Δ0 atg36Δ::kanMX4*) in the BY4742 genetic background [all from Thermo Scientific/Open Biosystems; #YSC1054, #YSC1021-550234 and #YSC1021-548143, respectively] were grown in YP medium (1% yeast extract, 2% peptone) [both from Fisher Scientific; #BP1422-2 and #BP1420-2, respectively] containing 0.2% glucose [#D16-10; Fisher Scientific] as carbon source. Cells were cultured at 30°C with rotational shaking at 200 rpm in Erlenmeyer flasks at a “flask volume/medium volume” ratio of 5:1.

### Identification of proteins by mass spectrometry

Solubilization of purified mitochondria for first-dimension blue native PAGE (1-D BN-PAGE), separation of the solubilized mitochondrial membrane protein supercomplexes by 1-D BN-PAGE, subsequent resolution of individual protein components of these supercomplexes by second-dimension tricine-SDS-PAGE (2-D SDS-PAGE) and following silver staining were carried out as previously described [34]. Protein bands were excised from the gel, reduced, alkylated and in-gel digested with trypsin [77]. The resulting peptides were separated by reverse phase high performance liquid chromatography coupled to mass spectrometry (RP-HPLC/MS) using an LTQ Orbitrap (#10145339; Thermo Scientific) [78]. The raw MS data file obtained by Xcalibur was analyzed using the Thermo Scientific Xcalibur Proteome Discoverer application (version 1.3) hereafter referred to as the Proteome Discoverer. The Proteome Discoverer was used to identify individual protein components of the isolated mitochondrial respiratory supercomplexes by comparing the raw data of mass spectra of digested fragments to the mass spectra of peptides within the Uniprot FASTA database. The analysis by the Proteome Discoverer coupled to the FASTA database was enabled by using the peak-finding search engine SEQUEST, which processes MS data using a peak-finding algorithm to search the raw data for generating a peak probability list with relative protein abundances.

### Analysis of lipids by mass spectrometry

Extraction of lipids from purified mitochondria, ER and PM and following mass spectrometric identification and quantitation of various lipid species were carried out as previously described [79]. Mass spectrometric analyses were performed with a Thermo OrbitrapVelos mass spectrometer equipped with a HESI-II ion source (#10145339; Thermo Scientific) operating at a flow rate of 5 μl/min. Numerous lipid species were separated by Fourier transform tandem mass spectrometry. Mass spectra were converted to an open format (mzXML, mzML) using the ProteoWizard MSConvert software freely available from http://proteowizard.source-forge.net/. The raw data were then imported into the open source software LipidXplorer (freely available from https://wiki.mpi-cbg.de/wiki/lipidx/index.php/-_Page) for the automated detection and quantitation of lipid species.

### Miscellaneous procedures

Chronological lifespan assay and pharmacological manipulation of chronological lifespan by addition of lithocholic acid (LCA) [Sigma; #L6250] were carried out as previously described [19]. The stock solution of LCA in water was made on the day of adding this compound to cell cultures. LCA was added to growth medium at the final concentration of 50 μM immediately following cell inoculation into the medium.

Fluorescence [18], immunofluorescence [18] and electron [80] microscopies followed by morphometric analyses of the resulting images were performed according to established procedures.

Cellular respiration assay [18], monitoring of the mitochondrial membrane potential [18], ROS measurement 81], immunodetection of carbonyl groups in oxidatively damaged mitochondrial proteins [81] and measurement of the frequencies of spontaneous mutations in mitochondrial DNA [56] were performed as previously described. Oxidatively damaged proteins and lipids in samples of purified mitochondria were determined with a Protein Carbonyl Assay Kit assay kit (#10005020; Cayman Chemical) and a PeroXOquant Quantitative Peroxide Assay Kit assay kit (#23285; Thermo Scientific Pierce), respectively, following the manufacturer's instructions.

Preparation of cellular extracts and a microanalytic biochemical assay for measuring ATP have been described elsewhere [82].

Subcellular fractionation of yeast [83] followed by isolation of mitochondria [84], ER [83] and cytosolic fraction [80] were performed according to established procedures. Yeast plasma membrane was isolated as previously described [85].

Protein concentration in samples of purified mitochondria was determined with an RC DC protein assay kit (#500-0122; Bio-Rad) following the manufacturer's instructions. SDS-PAGE and immunoblotting using a Trans-Blot SD semi-dry electrophoretic transfer system (#170-3940; Bio-Rad) were performed as previously described [86]. Blots were decorated with monoclonal antibodies raised against porin (#459500; Invitrogen) or polyclonal antisera raised against cytochrome *c* (kind gift of Dr. Roland Lill, Philipps Universität Marburg), cytochrome *c*1, subunit II of cytochrome *c* oxidase or alpha and beta subunits of the mitochondrial F1ATPase (all three antisera were kind gifts of Dr. Carla Koehler, University of California, Los Angeles). Antigen-antibody complexes were detected by enhanced chemilumines-cence using an Amersham ECL Plus Western Blotting Detection Reagents(#RPN2132; GE Healthcare).

State III and IV respiratory rates measurement using NADH as a respiratory substrate of external NADH:quinone oxidoreductase or succinate as a respiratory substrate of complex II, as well as uncoupled respiratory rates (UC) measurement in the presence of the uncoupler CCCP and a respiratory substrate (NADH or succinate) were performed by polarography of purified mitochondria according to established procedures [36]. The ratios of state III rate/state IV rate (*i.e.*, RCR), state III rate/UC rate and ADP/O (*i.e.*, the stoichiometry of coupled oxidation and phosphorylation) were calculated as previously described [37]. Enzymatic activities of NADH:decylu-biquinone oxidoreductase (NQR; an impaired in proton pumping across the IMM analog of the respiratory complex I in higher eukaryotes) [35], succinate:decy-lubiquinone-2,6-dichlorophenolindo-phenol oxido-reductase (the respiratory complex II) [87], ubiquinol:cytochrome *c* oxidoreductase (the respiratory complex III) [88], cytochrome *c* oxidase (the respiratory complex IV) [38] and F_1_F_0_-ATP synthase (the respiratory complex V) [39] were determined by established methods.

## SUPPLEMENTAL DATA



## References

[R1] Yen WL, Klionsky DJ (2008). How to live long and prosper: autophagy, mitochondria, and aging. Physiology.

[R2] Kanki T, Klionsky DJ, Okamoto K (2011). Mitochondria autophagy in yeast. Antioxid Redox Signal.

[R3] Vazquez-Martin A, Cufi S, Corominas-Faja B, Oliveras-Ferraros C, Vellon L, Menendez JA (2012). Mitochondrial fusion by pharmacological manipulation impedes somatic cell reprogramming to pluripotency: new insight into the role of mitophagy in cell stemness. Aging.

[R4] Bhatia-Kiššová I, Camougrand N (2010). Mitophagy in yeast: actors and physiological roles. FEMS Yeast Res.

[R5] May AI, Devenish RJ, Prescott M (2012). The many faces of mitochondrial autophagy: making sense of contrasting observations in recent research. Int J Cell Biol.

[R6] Kanki T, Klionsky DJ (2010). The molecular mechanism of mitochondria autophagy in yeast. Mol Microbiol.

[R7] Lee J, Giordano S, Zhang J (2012). Autophagy, mitochondria and oxidative stress: cross-talk and redox signalling. Biochem J.

[R8] Youle RJ, Narendra DP (2011). Mechanisms of mitophagy. Nat Rev Mol Cell Biol.

[R9] Hirota Y, Kang D, Kanki T (2012). The physiological role of mitophagy: new insights into phosphorylation events. Int J Cell Biol.

[R10] Tal R, Winter G, Ecker N, Klionsky DJ, Abeliovich H (2007). Aup1p, a yeast mitochondrial protein phosphatase homolog, is required for efficient stationary phase mitophagy and cell survival. J Biol Chem.

[R11] Journo D, Mor A, Abeliovich H (2009). Aup1-mediated regulation of Rtg3 during mitophagy. J Biol Chem.

[R12] Titorenko VI, Terlecky SR (2011). Peroxisome metabolism and cellular aging. Traffic.

[R13] Gey U, Czupalla C, Hoflack B, Rödel G, Krause-Buchholz U (2008). Yeast pyruvate dehydrogenase complex is regulated by a concerted activity of two kinases and two phosphatases. J Biol Chem.

[R14] Kurihara Y, Kanki T, Aoki Y, Hirota Y, Saigusa T, Uchiumi T, Kang D (2012). Mitophagy plays an essential role in reducing mitochondrial production of reactive oxygen species and mutation of mitochondrial DNA by maintaining mitochondrial quantity and quality in yeast. J Biol Chem.

[R15] Kanki T, Wang K, Cao Y, Baba M, Klionsky DJ (2009a). Atg32 is a mitochondrial protein that confers selectivity during mitophagy. Dev Cell.

[R16] Okamoto K, Kondo-Okamoto N, Ohsumi Y (2009). Mitochondria-anchored receptor Atg32 mediates degradation of mitochondria via selective autophagy. Dev Cell.

[R17] Kanki T, Wang K, Baba M, Bartholomew CR, Lynch-Day MA, Du Z, Geng J, Mao K, Yang Z, Yen WL, Klionsky DJ (2009b). A genomic screen for yeast mutants defective in selective mitochondria autophagy. Mol Biol Cell.

[R18] Goldberg AA, Bourque SD, Kyryakov P, Gregg C, Boukh-Viner T, Beach A, Burstein MT, Machkalyan G, Richard V, Rampersad S, Cyr D, Milijevic S, Titorenko VI (2009a). Effect of calorie restriction on the metabolic history of chronologically aging yeast. Exp Gerontol.

[R19] Goldberg AA, Richard VR, Kyryakov P, Bourque SD, Beach A, Burstein MT, Glebov A, Koupaki O, Boukh-Viner T, Gregg C, Juneau M, English AM, Thomas DY, Titorenko VI (2010). Chemical genetic screen identifies lithocholic acid as an anti-aging compound that extends yeast chronological life span in a TOR-independent manner, by modulating housekeeping longevity assurance processes. Aging.

[R20] Levine T (2004). Short-range intracellular trafficking of small molecules across endoplasmic reticulum junctions. Trends Cell Biol.

[R21] Lebiedzinska M, Szabadkai G, Jones AW, Duszynski J, Wieckowski MR (2009). Interactions between the endoplasmic reticulum, mitochondria, plasma membrane and other subcellular organelles. Int J Biochem Cell Biol.

[R22] Carrasco S, Meyer T (2011). STIM proteins and the endoplasmic reticulum-plasma membrane junctions. Annu Rev Biochem.

[R23] Elbaz Y, Schuldiner M (2011). Staying in touch: the molecular era of organelle contact sites. Trends Biochem Sci.

[R24] Friedman JR, Voeltz GK (2011). The ER in 3D: a multifunctional dynamic membrane network. Trends Cell Biol.

[R25] Osman C, Voelker DR, Langer T (2011). Making heads or tails of phospholipids in mitochondria. J Cell Biol.

[R26] Henry SA, Kohlwein SD, Carman GM (2012). Metabolism and regulation of glycerolipids in the yeast *Saccharomyces cerevisiae*. Genetics.

[R27] Rowland AA, Voeltz GK (2012). Endoplasmic reticulum-mitochondria contacts: function of the junction. Nat Rev Mol Cell Biol.

[R28] Motley AM, Nuttall JM, Hettema EH (2012). Pex3-anchored Atg36 tags peroxisomes for degradation in *Saccharomyces cerevisiae*. EMBO J.

[R29] Green DR, Galluzzi L, Kroemer G (2011). Mitochondria and the autophagy-inflammation-cell death axis in organismal aging. Science.

[R30] Fraenkel DG, Fraenkel DG (2011). Respiration.

[R31] Vonck J, Schäfer E (2009). Supramolecular organization of protein complexes in the mitochondrial inner membrane. Biochim Biophys Acta.

[R32] Lenaz G, Genova ML (2010). Structure and organization of mitochondrial respiratory complexes: a new understanding of an old subject. Antioxid Redox Signal.

[R33] Lenaz G, Genova ML (2012). Supramolecular organisation of the mitochondrial respiratory chain: a new challenge for the mechanism and control of oxidative phosphorylation. Adv Exp Med Biol.

[R34] Wittig I, Braun HP, Schägger H (2006). Blue native PAGE. Nat Protoc.

[R35] Schägger H, Pfeiffer K (2000). Supercomplexes in the respiratory chains of yeast and mammalian mitochondria. EMBO J.

[R36] Claypool SM, Oktay Y, Boontheung P, Loo JA, Koehler CM (2008). Cardiolipin defines the interactome of the major ADP/ATP carrier protein of the mitochondrial inner membrane. J Cell Biol.

[R37] Li Z, Graham BH (2012). Measurement of mitochondrial oxygen consumption using a Clark electrode. Methods Mol Biol.

[R38] Barrientos A (2002). In vivo and in organello assessment of OXPHOS activities. Methods.

[R39] Fontanesi F, Diaz F, Barrientos A (2009). Evaluation of the mitochondrial respiratory chain and oxidative phosphorylation system using yeast models of OXPHOS deficiencies. Curr Protoc Hum Genet.

[R40] Barros MH, Bandy B, Tahara EB, Kowaltowski AJ (2004). Higher respiratory activity decreases mitochondrial reactive oxygen release and increases life span in *Saccharomyces cerevisiae*. J Biol Chem.

[R41] Bonawitz ND, Rodeheffer MS, Shadel GS (2006). Defective mitochondrial gene expression results in reactive oxygen species-mediated inhibition of respiration and reduction of yeast life span. Mol Cell Biol.

[R42] Bonawitz ND, Chatenay-Lapointe M, Pan Y, Shadel GS (2007). Reduced TOR signaling extends chronological life span via increased respiration and upregulation of mitochondrial gene expression. Cell Metab.

[R43] Bonawitz ND, Shadel GS (2007). Rethinking the mitochondrial theory of aging: the role of mitochondrial gene expression in lifespan determination. Cell Cycle.

[R44] Pan Y, Shadel GS (2009). Extension of chronological life span by reduced TOR signaling requires down-regulation of Sch9p and involves increased mitochondrial OXPHOS complex density. Aging.

[R45] Pan Y, Schroeder EA, Ocampo A, Barrientos A, Shadel GS (2011). Regulation of yeast chronological life span by TORC1 via adaptive mitochondrial ROS signaling. Cell Metab.

[R46] Ocampo A, Liu J, Schroeder EA, Shadel GS, Barrientos A (2012). Mitochondrial respiratory thresholds regulate yeast chronological life span and its extension by caloric restriction. Cell Metab.

[R47] Lev S (2010). Non-vesicular lipid transport by lipid-transfer proteins and beyond. Nat Rev Mol Cell Biol.

[R48] Masoro EJ (2002). Caloric Restriction: A Key to Understanding and Modulating Aging.

[R49] Mair W, Dillin A (2008). Aging and survival: the genetics of life span extension by dietary restriction. Annu Rev Biochem.

[R50] Colman RJ, Anderson RM, Johnson SC, Kastman EK, Kosmatka KJ, Beasley TM, Allison DB, Cruzen C, Simmons HA, Kemnitz JW, Weindruch R (2009). Caloric restriction delays disease onset and mortality in rhesus monkeys. Science.

[R51] Leontieva OV, Blagosklonny MV (2011). Yeast-like chronological senescence in mammalian cells: phenomenon, mechanism and pharmacological suppression. Aging.

[R52] Fabrizio P, Wei M (2011). Conserved role of medium acidification in chronological senescence of yeast and mammalian cells. Aging.

[R53] Liao CY, Rikke BA, Johnson TE, Diaz V, Nelson JF (2010). Genetic variation in the murine lifespan response to dietary restriction: from life extension to life shortening. Aging Cell.

[R54] Mattison JA, Roth GS, Beasley TM, Tilmont EM, Handy AM, Herbert RL, Longo DL, Allison DB, Young JE, Bryant M, Barnard D, Ward WF, Qi W, Ingram DK, de Cabo R (2012). Impact of caloric restriction on health and survival in rhesus monkeys from the NIA study. Nature.

[R55] Beach A, Titorenko VI (2011). In search of housekeeping pathways that regulate longevity. Cell Cycle.

[R56] Burstein MT, Kyryakov P, Beach A, Richard VR, Koupaki O, Gomez-Perez A, Leonov A, Levy S, Noohi F, Titorenko (2012). Lithocholic acid extends longevity of chronologically aging yeast only if added at certain critical periods of their lifespan. Cell Cycle.

[R57] D'Autréaux B, Toledano MB (2007). ROS as signalling molecules: mechanisms that generate specificity in ROS homeostasis. Nat Rev Mol Cell Biol.

[R58] Giorgio M, Trinei M, Migliaccio E, Pelicci PG (2007). Hydrogen peroxide: a metabolic by-product or a common mediator of ageing signals?. Nat Rev Mol Cell Biol.

[R59] Barrientos A (2012). Complementary roles of mitochondrial respiration and ROS signaling on cellular aging and longevity. Aging.

[R60] Vandenabeele P, Galluzzi L, Vanden Berghe T, Kroemer G (2010). Molecular mechanisms of necroptosis: an ordered cellular explosion. Nat Rev Mol Cell Biol.

[R61] Carmona-Gutierrez D, Eisenberg T, Büttner S, Meisinger C, Kroemer G, Madeo F (2010). Apoptosis in yeast: triggers, pathways, subroutines. Cell Death Differ.

[R62] Eisenberg T, Carmona-Gutierrez D, Büttner S, Tavernarakis N, Madeo F (2010). Necrosis in yeast. Apoptosis.

[R63] Graef M, Nunnari J (2011). Mitochondria regulate autophagy by conserved signalling pathways. EMBO J.

[R64] Longo VD, Shadel GS, Kaeberlein M, Kennedy B (2012). Replicative and chronological aging in Saccharomyces cerevisiae. Cell Metab.

[R65] Yorimitsu T, Zaman S, Broach JR, Klionsky DJ (2007). Protein kinase A and Sch9 cooperatively regulate induction of autophagy in *Saccharomyces cerevisiae*. Mol Biol Cell.

[R66] Stephan JS, Yeh YY, Ramachandran V, Deminoff SJ, Herman PK (2009). The Tor and PKA signaling pathways independently target the Atg1/Atg13 protein kinase complex to control autophagy. Proc Natl Acad Sci USA.

[R67] Smets B, Ghillebert R, De Snijder P, Binda M, Swinnen E, De Virgilio C, Winderickx J (2010). Life in the midst of scarcity: adaptations to nutrient availability in *Saccharomyces cerevisiae*. Curr Genet.

[R68] Medvedik O, Lamming DW, Kim KD, Sinclair DA (2007). MSN2 and MSN4 link calorie restriction and TOR to sirtuin-mediated lifespan extension in *Saccharomyces cerevisiae*. PLoS Biol.

[R69] Goldberg AA, Bourque SD, Kyryakov P, Boukh-Viner T, Gregg C, Beach A, Burstein MT, Machkalyan G, Richard V, Rampersad S, Titorenko VI (2009b). A novel function of lipid droplets in regulating longevity. Biochem Soc Trans.

[R70] Gonzalvez F, Schug ZT, Houtkooper RH, MacKenzie ED, Brooks DG, Wanders RJ, Petit PX, Vaz FM, Gottlieb E (2008). Cardiolipin provides an essential activating platform for caspase-8 on mitochondria. J Cell Biol.

[R71] Sorice M, Manganelli V, Matarrese P, Tinari A, Misasi R, Malorni W, Garofalo T (2009). Cardiolipin-enriched raft-like microdomains are essential activating platforms for apoptotic signals on mitochondria. FEBS Lett.

[R72] Hailey DW, Rambold AS, Satpute-Krishnan P, Mitra K, Sougrat R, Kim PK, Lippincott-Schwartz J (2010). Mitochondria supply membranes for autophagosome biogenesis during starvation. Cell.

[R73] Knævelsrud H, Simonsen A (2012). Lipids in autophagy: constituents, signaling molecules and cargo with relevance to disease. Biochim Biophys Acta.

[R74] Rubinsztein DC, Shpilka T, Elazar Z (2012). Mechanisms of autophagosome biogenesis. Curr Biol.

[R75] Aoki Y, Kanki T, Hirota Y, Kurihara Y, Saigusa T, Uchiumi T, Kang D (2011). Phosphorylation of Serine 114 on Atg32 mediates mitophagy. Mol Biol Cell.

[R76] Mao K, Wang K, Zhao M, Xu T, Klionsky DJ (2011). Two MAPK-signaling pathways are required for mitophagy in *Saccharomyces cerevisiae*. J Cell Biol.

[R77] Shevchenko A, Jensen ON, Podtelejnikov AV, Sagliocco F, Mortensen P, Shevchenko A, Boucherie H, Mann M (1996). Linking genome and proteome by mass spectrometry: large-scale identification of yeast proteins from two dimensional gels. Proc Natl Acad Sci USA.

[R78] Michalski A, Damoc E, Hauschild JP, Lange O, Wieghaus A, Makarov A, Nagaraj N, Cox J, Mann M, Horning S (2011). Mass spectrometry-based proteomics using Q Exactive, a high-performance benchtop quadrupole Orbitrap mass spectrometer. Mol Cell Proteomics.

[R79] Bourque SD, Titorenko VI (2009). A quantitative assessment of the yeast lipidome using electrospray ionization mass spectrometry. J Vis Exp.

[R80] Guo T, Gregg C, Boukh-Viner T, Kyryakov P, Goldberg A, Bourque S, Banu F, Haile S, Milijevic S, San KH, Solomon J, Wong V, Titorenko VI (2007). A signal from inside the peroxisome initiates its division by promoting the remodeling of the peroxisomal membrane. J Cell Biol.

[R81] Kyryakov P, Beach A, Richard VR, Burstein MT, Leonov A, Levy S, Titorenko VI (2012). Caloric restriction extends yeast chronological lifespan by altering a pattern of age-related changes in trehalose concentration. Front Physiol.

[R82] Lin SS, Manchester JK, Gordon JI (2001). Enhanced gluconeogenesis and increased energy storage as hallmarks of aging in *Saccharomyces cerevisiae*. J Biol Chem.

[R83] Rieder SE, Emr SD, Bonifacino JS, Dasso M, Harford JB, Lippincott-Schwartz J, Yamada KM (2000). Isolation of subcellular fractions from the yeast *Saccharomyces cerevisiae*.

[R84] Gregg C, Kyryakov P, Titorenko VI (2009). Purification of mitochondria from yeast cells. J Vis Exp.

[R85] Panaretou B, Piper P (2006). Isolation of yeast plasma membranes. Methods Mol Biol.

[R86] Titorenko VI, Smith JJ, Szilard RK, Rachubinski RA (1998). Pex20p of the yeast *Yarrowia lipolytica* is required for the oligomerization of thiolase in the cytosol and for its targeting to the peroxisome. J Cell Biol.

[R87] Stroh A, Anderka O, Pfeiffer K, Yagi T, Finel M, Ludwig B, Schägger H (2004). Assembly of respiratory complexes I, III, and IV into NADH oxidase supercomplex stabilizes complex I in *Paracoccus denitrificans*. J Biol Chem.

[R88] Krause F, Scheckhuber CQ, Werner A, Rexroth S, Reifschneider NH, Dencher NA, Osiewacz HD (2004). Supramolecular organization of cytochrome *c* oxidase- and alternative oxidase-dependent respiratory chains in the filamentous fungus *Podospora anserina*. J Biol Chem.

