# Supplementary material for: Macromitophagy is a longevity assurance process that in chronologically aging yeast limited in calorie supply sustains functional mitochondria and maintains cellular lipid homeostasis
Source: Aging (Albany NY). 2013 Mar 30;5(4):234–69. doi: 10.18632/aging.100547 (PMC3651518; doi:10.18632/aging.100547)
Supplement: Supplementary file 1 [file aging-05-234-s001.pdf]

SUPPLEMENTAL DATA

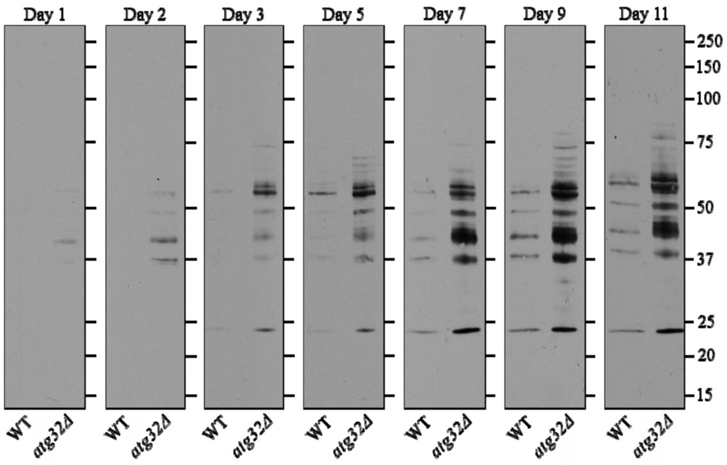

Figure S1. Under CR conditions, the atg32Δ mutation elevates the extent of oxidative damage to mitochondrial proteins. WT and atg32Δ strains were cultured in the nutrient-rich YP medium initially containing 0.2% glucose. Immunodetection of carbonyl groups in oxidatively damaged proteins of purified mitochondria was performed as described in Methods.

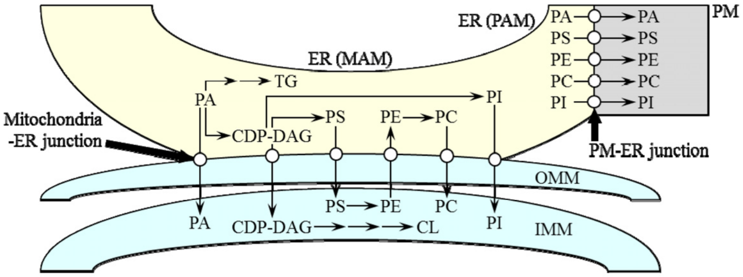

Figure S2. Lipid synthesis in the ER and mitochondria and lipid transport via mitochondria-ER and PM-ER junctions. After being synthesized in the mitochondria-associated membrane (MAM) domain of the ER, cytidine diphosphate-diacylglycerol (CDP-DAG) and phosphatidylserine (PS) are transported via mitochondria-ER junctions to the outer mitochondrial membrane (OMM). Following their translocation from the OMM to the inner mitochondrial membrane (IMM), CDP-DAG and PS are used as substrates for the synthesis of cardiolipin (CL) and phosphatidylethanolamine (PE), respectively. PE is then transported through mitochondria-ER junctions to the MAM domain of the ER, where it is converted to phosphatidylcholine (PC). After being formed in the MAM, PC is translocated via mitochondria-ER junctions to mitochondria, which are incapable of synthesizing this glycerophospholipid. The glycerophospholipids phosphatidic acid (PA) and phosphatidylinositol (PI) are also synthesized in the MAM domain of the ER, from which they are delivered through mitochondria-ER junctions to mitochondria known to lack enzymes required for the synthesis of these two glycerophospholipids. After being synthesized within the ER and inner mitochondrial membranes, lipids can be translocated via the PM-ER junctions from the PM-associated membrane (PAM) domain of the ER to the plasma membrane (PM). Unlike glycerophospholipids, various molecular forms of the neutral lipids triacylglycerols (TG) cannot be moved from the site of their synthesis in the ER to either mitochondria or the PM. See text for details.

Table S1. List of individual protein components of digitonin-solubilized mitochondrial membrane protein supercomplexes separated by 1-D BN-PAGE, resolved by 2-D SDS-PAGE and stained by silver. Following silver staining, protein bands were excised from the gel, reduced, alkylated and in-gel digested with trypsin. The resulting peptides were separated by RP-HPLC/MS using an LTQ Orbitrap. The raw MS data file obtained by Xcalibur was analyzed using the Thermo Scientific Xcalibur Proteome Discoverer application (version 1.3), which was used to identify individual protein components of the isolated mitochondrial respiratory supercomplexes by comparing the raw data of mass spectra of digested fragments to the mass spectra of peptides within the Uniprot FASTA database. The analysis by the Thermo Scientific Xcalibur Proteome Discoverer coupled to the FASTA database was enabled by using the peak-finding search engine SEQUEST, which processes MS data using a peak-finding algorithm to search the raw data for generating a peak probability list with relative protein abundances.

Protein name	Protein description	NCBI accession number	Score	Sequence coverage (%)	Number of identified peptides	MW (kDa)	Calculated pI
**Wild-type strain**							
**Protein supercomplex 1**							
Aac2p	Major ADP/ATP carrier of the mitochondrial inner membrane	P18239	103.33	30.19%	10	34.4	9.79
Por1p	Mitochondrial porin (voltage-dependent anion channel)	P04840	251.47	43.46%	12	30.4	7.93
Ndi1p	NADH:ubiquinone oxidoreductase	P32340	177.99	35.67%	19	57.2	9.44
Nde1p	Mitochondrial external NADH dehydrogenase	P40215	56.52	34.64%	25	62.7	9.28
Pda1p	E1 alpha subunit of the pyruvate dehydrogenase complex	P16387	99.69	26.43%	15	46.3	8.1
Pdb1p	E1 beta subunit of the pyruvate dehydrogenase complex	P32473	84.43	34.43%	9	40	5.3
Lat1p	Dihydrolipoamide acetyltransferase component (E2) of the pyruvate dehydrogenase complex	P12695	118.56	26.56%	11	51.8	7.8
Lpd1p	Dihydrolipoamide dehydrogenase component (E3) of the pyruvate dehydrogenase complex	P09624	253.54	51.70%	27	54	8.03

Sdh1p	Flavoprotein subunit of succinate dehydrogenase (respiratory complex II)	Q00711	112.55	38.59%	24	70.2	7.5
Sdh2p	Iron-sulfur subunit of succinate dehydrogenase (respiratory complex II)	P21801	212.02	31.20%	12	30.2	8.82
Sdh3p	Cytochrome b subunit of succinate dehydrogenase (respiratory complex II)	P33421	97.47	22.73%	6	22.1	10.23
Sdh4p	Membrane anchor subunit of succinate dehydrogenase (respiratory complex II)	P37298	129.27	24.31%	6	20.2	8.9
Cyt1p	Cytochrome b-c1 complex (respiratory complex III), cytochrome c1 component	P07143	49.94	27.83%	5	34	8.12
Qcr1p	Cytochrome b-c1 complex (respiratory complex III), subunit 1	P07256	154.4	32.82%	19	50.2	7.3
Qcr2p	Cytochrome b-c1 complex (respiratory complex III), subunit 2	P07257	188.18	54.62%	26	40.5	7.96
Qcr7p	Cytochrome b-c1 complex (respiratory complex III), subunit 7	P00128	106.36	22.83%	5	14.6	5.88
Qcr8p	Cytochrome b-c1 complex (respiratory complex III), subunit 8	P08525	92.45	24.47%	3	11	9.7
Rip1p	Cytochrome b-c1 complex (respiratory complex III), ubiquinol-cytochrome-c reductase	P08067	68.73	18.60%	5	23.3	8.07

Atp1p	Mitochondrial F1F0 ATP synthase (respiratory complex V), alpha subunit	P07251	260.88	23.67%	15	58.6	9.04
Atp2p	Mitochondrial F1F0 ATP synthase (respiratory complex V), beta subunit	P00830	270.34	38.94%	20	54.8	5.71
Atp3p	Mitochondrial F1F0 ATP synthase (respiratory complex V), gamma subunit	P38077	207.02	47.91%	20	34.3	9.31
Atp4p	Mitochondrial F1F0 ATP synthase (respiratory complex V), b subunit	P05626	135.52	55.74%	18	26.9	9.13
Atp5p	Mitochondrial F1F0 ATP synthase (respiratory complex V), subunit 5	P09457	197.56	86.32%	21	22.8	9.57
Atp7p	Mitochondrial F1F0 ATP synthase (respiratory complex V), d subunit	P30902	256.15	66.67%	20	19.8	8.94
Atp17p	Mitochondrial F1F0 ATP synthase (respiratory complex V), f subunit	Q06405	91.74	41.58%	5	11.3	10.1
**Protein supercomplex 2**							
Aac2p	Major ADP/ATP carrier of the mitochondrial inner membrane	P18239	92.94	38.99%	15	34.4	9.79
Por1p	Mitochondrial porin (voltage-dependent anion channel)	P04840	199.73	39.22%	10	30.4	7.93
Kgd1p	Component of the mitochondrial alpha-ketoglutarate dehydrogenase complex	P20967	409.94	56.21%	53	114.3	7.21

	Cytochrome b-c1 complex (respiratory complex III), cytochrome c1 component	P07143	35.71	27.83%	5	34	8.12
Qcr1p	Cytochrome b-c1 complex (respiratory complex III), subunit 1	P07256	636.69	36.32%	20	50.2	7.3
Qcr2p	Cytochrome b-c1 complex (respiratory complex III), subunit 2	P07257	248.78	67.12%	33	40.5	7.96
Qcr7p	Cytochrome b-c1 complex (respiratory complex III), subunit 7	P00128	122.57	22.83%	5	14.6	5.88
Qcr8p	Cytochrome b-c1 complex (respiratory complex III), subunit 8	P08525	26.43	24.47%	4	11	9.7
Rip1p	Cytochrome b-c1 complex (respiratory complex III), ubiquinol-cytochrome-c reductase	P08067	61.51	28.37%	6	23.3	8.07
**Protein supercomplex 3**							
Cyt1p	Cytochrome b-c1 complex (respiratory complex III), cytochrome c1 component	P07143	40.02	22.98%	6	34	8.12
Qcr1p	Cytochrome b-c1 complex (respiratory complex III), subunit 1	P07256	124.86	34.14%	17	50.2	7.3
Qcr2p	Cytochrome b-c1 complex (respiratory complex III), subunit 2	P07257	156.88	57.61%	25	40.5	7.96
Qcr7p	Cytochrome b-c1 complex (respiratory complex III), subunit 7	P00128	142.84	51.97%	7	14.6	5.88

Qcr8p	Cytochrome b-c1 complex (respiratory complex III), subunit 8	P08525	40.05	24.47%	4	11	9.7
Rip1p	Cytochrome b-c1 complex (respiratory complex III), ubiquinol-cytochrome-c reductase	P08067	54.99	25.58%	6	23.3	8.07
Atp1p	Mitochondrial F1F0 ATP synthase (respiratory complex V), alpha subunit	P07251	174.59	28.26%	21	58.6	9.04
Atp2p	Mitochondrial F1F0 ATP synthase (respiratory complex V), beta subunit	P00830	129.72	55.77%	23	54.8	5.71
Atp3p	Mitochondrial F1F0 ATP synthase (respiratory complex V), gamma subunit	P38077	143.47	32.80%	12	34.3	9.31
Atp4p	Mitochondrial F1F0 ATP synthase (respiratory complex V), b subunit	P05626	254.25	50.82%	14	26.9	9.13
Atp5p	Mitochondrial F1F0 ATP synthase (respiratory complex V), subunit 5	P09457	91.52	55.19%	11	22.8	9.57
Atp7p	Mitochondrial F1F0 ATP synthase (respiratory complex V), d subunit	P30902	166.24	40.23%	11	19.8	8.94
Atp17p	Mitochondrial F1F0 ATP synthase (respiratory complex V), f subunit	Q06405	83.21	32.67%	4	11.3	10.1

**Protein supercomplex 4**							
Aac2p	Major ADP/ATP carrier of the mitochondrial inner membrane	P18239	92.83	29.25%	10	34.4	9.79
Atp1p	Mitochondrial F1F0 ATP synthase (respiratory complex V), alpha subunit	P07251	255.88	28.07%	22	58.6	9.04
Atp2p	Mitochondrial F1F0 ATP synthase (respiratory complex V), beta subunit	P00830	292.07	31.70%	18	54.8	5.71
Atp3p	Mitochondrial F1F0 ATP synthase (respiratory complex V), gamma subunit	P38077	615.01	41.80%	16	34.3	9.31
Atp4p	Mitochondrial F1F0 ATP synthase (respiratory complex V), b subunit	P05626	90.24	40.57%	12	26.9	9.13
Atp5p	Mitochondrial F1F0 ATP synthase (respiratory complex V), subunit 5	P09457	225.76	66.51%	12	22.8	9.57
Atp7p	Mitochondrial F1F0 ATP synthase (respiratory complex V), d subunit	P30902	125.76	51.15%	14	19.8	8.94
**Protein supercomplex 5**							
Yme2p	Integral inner mitochondrial membrane protein with a role in maintaining mitochondrial nucleoid structure and number	P32843	194.31	35.65%	32	96.6	8.92
**Protein supercomplex 6**							
Aac2p	Major ADP/ATP carrier of the mitochondrial inner membrane	P18239	184.97	51.26%	24	34.4	9.79

Atp1p	Mitochondrial F1F0 ATP synthase (respiratory complex V), alpha subunit	P07251	173.98	39.08%	28	58.6	9.04
Atp2p	Mitochondrial F1F0 ATP synthase (respiratory complex V), beta subunit	P00830	106.04	42.66%	21	54.8	5.71
Atp3p	Mitochondrial F1F0 ATP synthase (respiratory complex V), gamma subunit	P38077	162.87	26.05%	9	34.3	9.31
Atp4p	Mitochondrial F1F0 ATP synthase (respiratory complex V), b subunit	P05626	173.15	50.82%	15	26.9	9.13
Atp5p	Mitochondrial F1F0 ATP synthase (respiratory complex V), subunit 5	P09457	108.74	48.58%	11	22.8	9.57
Atp7p	Mitochondrial F1F0 ATP synthase (respiratory complex V), d subunit	P30902	101.36	58.05%	16	19.8	8.94
**Protein supercomplex 7**							
Cyt1p	Cytochrome b-c1 complex (respiratory complex III), cytochrome c1 component	P07143	36.2	22.65%	5	34	8.12
Qcr1p	Cytochrome b-c1 complex (respiratory complex III), subunit 1	P07256	242.73	39.17%	20	50.2	7.3
Qcr2p	Cytochrome b-c1 complex (respiratory complex III), subunit 2	P07257	178.59	74.46%	33	40.5	7.96

Qcr7p	Cytochrome b-c1 complex (respiratory complex III), subunit 7	P00128	131.52	22.83%	5	14.6	5.88
Qcr8p	Cytochrome b-c1 complex (respiratory complex III), subunit 8	P08525	26.82	24.47%	4	11	9.7
Rip1p	Cytochrome b-c1 complex (respiratory complex III), ubiquinol-cytochrome-c reductase	P08067	77.37	36.28%	10	23.3	8.07
Cox4p	Cytochrome c oxidase (respiratory complex IV), subunit 4	P04037	14.78	31.61%	3	17.1	6.9
Cox5Ap	Cytochrome c oxidase (respiratory complex IV), subunit 5A	P00424	160.43	47.71%	11	17.1	9.82
Cox5Bp	Cytochrome c oxidase (respiratory complex IV), subunit 5B	P00425	103.71	28.48%	5	17.2	9.8
Cox6p	Cytochrome c oxidase (respiratory complex IV), subunit 6	P00427	26.7	33.11%	8	17.3	6.05
Atp1p	Mitochondrial F1F0 ATP synthase (respiratory complex V), alpha subunit	P07251	180.84	48.44%	32	58.6	9.04
Atp2p	Mitochondrial F1F0 ATP synthase (respiratory complex V), beta subunit	P00830	158.5	40.51%	20	54.8	5.71
Atp3p	Mitochondrial F1F0 ATP synthase (respiratory complex V), gamma subunit	P38077	387	30.55%	10	34.3	9.31

Atp4p	Mitochondrial F1F0 ATP synthase (respiratory complex V), b subunit	P05626	95.35	45.90%	18	26.9	9.13
Atp5p	Mitochondrial F1F0 ATP synthase (respiratory complex V), subunit 5	P09457	288.43	66.98%	14	22.8	9.57
Atp7p	Mitochondrial F1F0 ATP synthase (respiratory complex V), d subunit	P30902	196.03	44.25%	13	19.8	8.94
**Protein supercomplex 8**							
Ald4p	Mitochondrial aldehyde dehydrogenase	P46367	531.66	52.60%	24	56.7	6.74
Por1p	Mitochondrial porin (voltage-dependent anion channel)	P04840	114.07	42.05%	11	30.4	7.93
Cyt1p	Cytochrome b-c1 complex (respiratory complex III), cytochrome c1 component	P07143	32.23	29.77%	6	34	8.12
Qcr1p	Cytochrome b-c1 complex (respiratory complex III), subunit 1	P07256	206.37	30.85%	17	50.2	7.3
Qcr2p	Cytochrome b-c1 complex (respiratory complex III), subunit 2	P07257	152.45	48.10%	21	40.5	7.96
Qcr7p	Cytochrome b-c1 complex (respiratory complex III), subunit 7	P00128	149.7	36.22%	6	14.6	5.88
Qcr8p	Cytochrome b-c1 complex (respiratory complex III), subunit 8	P08525	92.45	24.47%	3	11	9.7

Rip1p	Cytochrome b-c1 complex (respiratory complex III), ubiquinol-cytochrome-c reductase	P08067	36.14	33.95%	7	23.3	8.07
Atp1p	Mitochondrial F1F0 ATP synthase (respiratory complex V), alpha subunit	P07251	666.64	36.15%	27	58.6	9.04
Atp2p	Mitochondrial F1F0 ATP synthase (respiratory complex V), beta subunit	P00830	273.89	44.03%	20	54.8	5.71
Atp3p	Mitochondrial F1F0 ATP synthase (respiratory complex V), gamma subunit	P38077	432.23	40.19%	14	34.3	9.31
Atp4p	Mitochondrial F1F0 ATP synthase (respiratory complex V), b subunit	P05626	203.16	47.13%	15	26.9	9.13
Atp5p	Mitochondrial F1F0 ATP synthase (respiratory complex V), subunit 5	P09457	148.55	49.53%	11	22.8	9.57
Atp7p	Mitochondrial F1F0 ATP synthase (respiratory complex V), d subunit	P30902	112.24	40.80%	9	19.8	8.94
Idh1p	Subunit of mitochondrial NAD(+)-dependent isocitrate dehydrogenase	P28834	415.06	47.22%	19	39.3	9
Idh2p	Subunit of mitochondrial NAD(+)-dependent isocitrate dehydrogenase	P28241	238.75	39.84%	13	39.7	8.69

***atg32Δ* strain**							
**Protein supercomplex 1**							
Aac2p	Major ADP/ATP carrier of the mitochondrial inner membrane	P18239	142.9	31.45%	13	34.4	9.79
Por1p	Mitochondrial porin (voltage-dependent anion channel)	P04840	135.62	43.46%	12	30.4	7.93
Lat1p	Dihydrolipoamide acetyltransferase component (E2) of the pyruvate dehydrogenase complex	P12695	71.58	26.97%	14	51.8	7.8
Lpd1p	Dihydrolipoamide dehydrogenase component (E3) of the pyruvate dehydrogenase complex	P09624	171.86	37.07%	21	54	8.03
Cyt1p	Cytochrome b-c1 complex (respiratory complex III), cytochrome c1 component	P07143	68.38	31.07%	7	34	8.12
Qcr1p	Cytochrome b-c1 complex (respiratory complex III), subunit 1	P07256	216.4	34.79%	18	50.2	7.3
Qcr2p	Cytochrome b-c1 complex (respiratory complex III), subunit 2	P07257	134.35	56.25%	24	40.5	7.96
Qcr7p	Cytochrome b-c1 complex (respiratory complex III), subunit 7	P00128	160.32	36.22%	6	14.6	5.88
Qcr8p	Cytochrome b-c1 complex (respiratory complex III), subunit 8	P08525	26.43	24.47%	4	11	9.7
Rip1p	Cytochrome b-c1 complex (respiratory complex III), ubiquinol-cytochrome-c reductase	P08067	56.97	29.77%	7	23.3	8.07

Atp1p	Mitochondrial F1F0 ATP synthase (respiratory complex V), alpha subunit	P07251	293.31	28.26%	21	58.6	9.04
Atp2p	Mitochondrial F1F0 ATP synthase (respiratory complex V), beta subunit	P00830	329.47	43.84%	22	54.8	5.71
Atp3p	Mitochondrial F1F0 ATP synthase (respiratory complex V), gamma subunit	P38077	438.8	36.33%	13	34.3	9.31
Atp4p	Mitochondrial F1F0 ATP synthase (respiratory complex V), b subunit	P05626	298.9	62.30%	24	26.9	9.13
Atp5p	Mitochondrial F1F0 ATP synthase (respiratory complex V), subunit 5	P09457	573.45	75.47%	16	22.8	9.57
Atp7p	Mitochondrial F1F0 ATP synthase (respiratory complex V), d subunit	P30902	190.75	72.99%	15	19.8	8.94
**Protein supercomplex 2**							
Aac2p	Major ADP/ATP carrier of the mitochondrial inner membrane	P18239	167.8	34.91%	14	34.4	9.79
Por1p	Mitochondrial porin (voltage-dependent anion channel)	P04840	296.37	50.18%	14	30.4	7.93
Cyt1p	Cytochrome b-c1 complex (respiratory complex III), cytochrome c1 component	P07143	61.72	27.83%	5	34	8.12
Qcr1p	Cytochrome b-c1 complex (respiratory complex III), subunit 1	P07256	110.93	39.17%	20	50.2	7.3

Qcr2p	Cytochrome b-c1 complex (respiratory complex III), subunit 2	P07257	152.7	54.08%	24	40.5	7.96
Qcr7p	Cytochrome b-c1 complex (respiratory complex III), subunit 7	P00128	101.24	36.22%	6	14.6	5.88
Qcr8p	Cytochrome b-c1 complex (respiratory complex III), subunit 8	P08525	13.41	21.28%	2	11	9.7
Rip1p	Cytochrome b-c1 complex (respiratory complex III), ubiquinol-cytochrome-c reductase	P08067	52.77	46.05%	11	23.3	8.07
**Protein supercomplex 3**							
Atp1p	Mitochondrial F1F0 ATP synthase (respiratory complex V), alpha subunit	P07251	109.69	35.78%	25	58.6	9.04
Atp2p	Mitochondrial F1F0 ATP synthase (respiratory complex V), beta subunit	P00830	512.23	38.16%	22	54.8	5.71
Atp3p	Mitochondrial F1F0 ATP synthase (respiratory complex V), gamma subunit	P38077	156.34	36.33%	12	34.3	9.31
Atp4p	Mitochondrial F1F0 ATP synthase (respiratory complex V), b subunit	P05626	242.42	58.20%	22	26.9	9.13
Atp5p	Mitochondrial F1F0 ATP synthase (respiratory complex V), subunit 5	P09457	124.41	53.30%	11	22.8	9.57

Atp7p	Mitochondrial F1F0 ATP synthase (respiratory complex V), d subunit	P30902	348.32	79.89%	20	19.8	8.94
**Protein supercomplex 4**							
Aac2p	Major ADP/ATP carrier of the mitochondrial inner membrane	P18239	91.45	33.02%	11	34.4	9.79
**Protein supercomplex 6**							
Aac2p	Major ADP/ATP carrier of the mitochondrial inner membrane	P18239	176.99	45.60%	18	34.4	9.79
**Protein supercomplex 7**							
Atp1p	Mitochondrial F1F0 ATP synthase (respiratory complex V), alpha subunit	P07251	193.91	43.85%	27	58.6	9.04
Atp2p	Mitochondrial F1F0 ATP synthase (respiratory complex V), beta subunit	P00830	102.93	40.12%	17	54.8	5.71
Atp3p	Mitochondrial F1F0 ATP synthase (respiratory complex V), gamma subunit	P38077	138.31	36.33%	13	34.3	9.31
Atp4p	Mitochondrial F1F0 ATP synthase (respiratory complex V), b subunit	P05626	110.07	40.57%	14	26.9	9.13
Atp5p	Mitochondrial F1F0 ATP synthase (respiratory complex V), subunit 5	P09457	345.82	82.55%	19	22.8	9.57
Atp7p	Mitochondrial F1F0 ATP synthase (respiratory complex V), d subunit	P30902	198.09	83.91%	24	19.8	8.94

**Protein supercomplex 8**							
Ald4p	Mitochondrial aldehyde dehydrogenase	P46367	211.93	51.25%	21	56.7	6.74
Por1p	Mitochondrial porin (voltage-dependent anion channel)	P04840	141.79	40.64%	10	30.4	7.93
Idh1p	Subunit of mitochondrial NAD(+)-dependent isocitrate dehydrogenase	P28834	367.21	43.06%	16	39.3	9
Idh2p	Subunit of mitochondrial NAD(+)-dependent isocitrate dehydrogenase	P28241	580.69	40.65%	14	39.7	8.69
